# *ADAT3* variants disrupt the activity of the ADAT tRNA deaminase complex and impair neuronal migration

**DOI:** 10.1093/brain/awaf109

**Published:** 2025-03-22

**Authors:** Jordi Del-Pozo-Rodriguez, Peggy Tilly, Romain Lecat, Hugo Rolando Vaca, Laureline Mosser, Elena Brivio, Till Balla, Marina Vitoria Gomes, Elizabeth Ramos-Morales, Noémie Schwaller, Thalia Salinas-Giegé, Grace VanNoy, Eleina M England, Alysia Kern Lovgren, Melanie O’Leary, Maya Chopra, Naomi Meave Ojeda, Mehran Beiraghi Toosi, Atieh Eslahi, Masoome Alerasool, Majid Mojarrad, Lynn S Pais, Rebecca C Yeh, Dustin L Gable, Mais O Hashem, Firdous Abdulwahab, Muath Rakiz Alqurashi, Loai Z Sbeih, Omar Abu Adas Blanco, Renad Abu Khater, Gabriela Oprea, Aboulfazl Rad, Hamad Alzaidan, Hesham Aldhalaan, Ehab Tous, Afaf Alsagheir, Mohammed Alowain, Abdullah Tamim, Khowlah Alfayez, Amal Alhashem, Aisha Alnuzha, Mona Kamel, Bashayer S Al-Awam, Walaa Elnaggar, Nihal Almenabawy, Anne O'Donnell-Luria, Jennifer E Neil, Joseph G Gleeson, Christopher A Walsh, Fowzan S Alkuraya, Lama AlAbdi, Nour Elkhateeb, Laila Selim, Siddharth Srivastava, Danny D Nedialkova, Laurence Drouard, Christophe Romier, Efil Bayam, Juliette D Godin

**Affiliations:** IGBMC, Institut de Génétique et de Biologie Moléculaire et Cellulaire, Illkirch 67400, France; CNRS, Centre National de la Recherche Scientifique, UMR 7104, Illkirch 67400, France; INSERM, Institut National de la Santé et de la Recherche Médicale, UMR-S 1258, Illkirch 67400, France; IGBMC UMR 7104-UMR-S 1258, Université de Strasbourg, Illkirch 67400, France; IGBMC, Institut de Génétique et de Biologie Moléculaire et Cellulaire, Illkirch 67400, France; CNRS, Centre National de la Recherche Scientifique, UMR 7104, Illkirch 67400, France; INSERM, Institut National de la Santé et de la Recherche Médicale, UMR-S 1258, Illkirch 67400, France; IGBMC UMR 7104-UMR-S 1258, Université de Strasbourg, Illkirch 67400, France; IGBMC, Institut de Génétique et de Biologie Moléculaire et Cellulaire, Illkirch 67400, France; CNRS, Centre National de la Recherche Scientifique, UMR 7104, Illkirch 67400, France; INSERM, Institut National de la Santé et de la Recherche Médicale, UMR-S 1258, Illkirch 67400, France; IGBMC UMR 7104-UMR-S 1258, Université de Strasbourg, Illkirch 67400, France; IGBMC, Institut de Génétique et de Biologie Moléculaire et Cellulaire, Illkirch 67400, France; CNRS, Centre National de la Recherche Scientifique, UMR 7104, Illkirch 67400, France; INSERM, Institut National de la Santé et de la Recherche Médicale, UMR-S 1258, Illkirch 67400, France; IGBMC UMR 7104-UMR-S 1258, Université de Strasbourg, Illkirch 67400, France; Institut de biologie moléculaire des plantes, CNRS, Université de Strasbourg, Strasbourg 67084, France; IGBMC, Institut de Génétique et de Biologie Moléculaire et Cellulaire, Illkirch 67400, France; CNRS, Centre National de la Recherche Scientifique, UMR 7104, Illkirch 67400, France; INSERM, Institut National de la Santé et de la Recherche Médicale, UMR-S 1258, Illkirch 67400, France; IGBMC UMR 7104-UMR-S 1258, Université de Strasbourg, Illkirch 67400, France; Max Planck Institute of Biochemistry, Martinsried 82152, Germany; IGBMC, Institut de Génétique et de Biologie Moléculaire et Cellulaire, Illkirch 67400, France; CNRS, Centre National de la Recherche Scientifique, UMR 7104, Illkirch 67400, France; INSERM, Institut National de la Santé et de la Recherche Médicale, UMR-S 1258, Illkirch 67400, France; IGBMC UMR 7104-UMR-S 1258, Université de Strasbourg, Illkirch 67400, France; IGBMC, Institut de Génétique et de Biologie Moléculaire et Cellulaire, Illkirch 67400, France; CNRS, Centre National de la Recherche Scientifique, UMR 7104, Illkirch 67400, France; INSERM, Institut National de la Santé et de la Recherche Médicale, UMR-S 1258, Illkirch 67400, France; IGBMC UMR 7104-UMR-S 1258, Université de Strasbourg, Illkirch 67400, France; IGBMC, Institut de Génétique et de Biologie Moléculaire et Cellulaire, Illkirch 67400, France; CNRS, Centre National de la Recherche Scientifique, UMR 7104, Illkirch 67400, France; INSERM, Institut National de la Santé et de la Recherche Médicale, UMR-S 1258, Illkirch 67400, France; IGBMC UMR 7104-UMR-S 1258, Université de Strasbourg, Illkirch 67400, France; Institut de biologie moléculaire des plantes, CNRS, Université de Strasbourg, Strasbourg 67084, France; Broad Center for Mendelian Genomics, Broad Institute of MIT and Harvard, Cambridge, MA 02142, USA; Broad Center for Mendelian Genomics, Broad Institute of MIT and Harvard, Cambridge, MA 02142, USA; Broad Center for Mendelian Genomics, Broad Institute of MIT and Harvard, Cambridge, MA 02142, USA; Broad Center for Mendelian Genomics, Broad Institute of MIT and Harvard, Cambridge, MA 02142, USA; Department of Neurology, Boston Children’s Hospital, Boston, MA 02115, USA; Rosamund Stone Zander Translational Neuroscience Center, Boston Children’s Hospital, Boston, MA 02115, USA; Department of Neurosciences, University of California San Diego, La Jolla, CA 92093, USA; Rady Children’s Hospital, Rady Children’s Institute for Genomic Medicine, San Diego, CA 92128, USA; Department of Pediatrics, School of Medicine, Mashhad University of Medical Sciences, Mashhad 91778 99191, Iran; Department of Medical Genetics, Faculty of Medicine, Mashhad University of Medical Sciences, Mashhad 91778 99191, Iran; Department of Medical Genetics, Faculty of Medicine, Mashhad University of Medical Sciences, Mashhad 91778 99191, Iran; Genetic Foundation of Khorasan Razavi, Mashhad 91778 99191, Iran; Department of Medical Genetics, Faculty of Medicine, Mashhad University of Medical Sciences, Mashhad 91778 99191, Iran; Genetic Foundation of Khorasan Razavi, Mashhad 91778 99191, Iran; Broad Center for Mendelian Genomics, Broad Institute of MIT and Harvard, Cambridge, MA 02142, USA; Division of Genetics and Genomics, Boston Children’s Hospital, Harvard Medical School, Boston, MA 02115, USA; Division of Genetics and Genomics, Boston Children’s Hospital, Harvard Medical School, Boston, MA 02115, USA; Child Neurology Residency Training Program, Boston Children’s Hospital, Boston, MA 02115, USA; Department of Translational Genomics, Genomic Medicine Centre of Excellence, King Faisal Specialist Hospital and Research Center, Riyadh 11564, Saudi Arabia; Department of Translational Genomics, Genomic Medicine Centre of Excellence, King Faisal Specialist Hospital and Research Center, Riyadh 11564, Saudi Arabia; Child Neurologist Department, Al Khalidi Hospital, Amman 11183, Jordan; MedLabs, Amman 11183 Jordan; MedLabs, Amman 11183 Jordan; MedLabs, Amman 11183 Jordan; Arcensus GmbH, Rostock 18119, Germany; Arcensus GmbH, Rostock 18119, Germany; Department of Medical Genomics, Centre for Genomic Medicine, King Faisal Specialist Hospital and Research Center, Riyadh 11564, Saudi Arabia; Neuroscience Center, King Faisal Specialist Hospital and Research Center, Riyadh 11564, Saudi Arabia; Neuroscience Center, King Faisal Specialist Hospital and Research Center, Riyadh 11564, Saudi Arabia; Department of Pediatrics, King Faisal Specialist Hospital & Research Centre, Riyadh 11564, Saudi Arabia; College of Medicine, Alfaisal University, Riyadh 11533, Saudi Arabia; Department of Medical Genomics, Centre for Genomic Medicine, King Faisal Specialist Hospital and Research Center, Riyadh 11564, Saudi Arabia; College of Medicine, Alfaisal University, Riyadh 11533, Saudi Arabia; Department of Pediatrics, King Faisal Specialist Hospital and Research Center, Jeddah 23433, Saudi Arabia; Department of Pediatrics, Prince Sultan Military Medical Center, Riyadh 12233, Saudi Arabia; College of Medicine, Alfaisal University, Riyadh 11533, Saudi Arabia; Seha Virtual Hospital, Ministry of Health, Riyadh 12382, Saudi Arabia; Department of Genetics and Metabolics, King Fahad Specialist Hospital, Dammam 32253, Saudi Arabia; Department of Pediatrics, Pediatric Neurology Unit, King Salman Medical City, Madinah 7815, Saudi Arabia; Department of Pediatrics, Pediatric Neurology Unit, King Salman Medical City, Madinah 7815, Saudi Arabia; Department of Pediatrics, Pediatric Neurology and Metabolic Medicine Unit, Cairo University, Cairo 11628, Egypt; Department of Pediatrics, College of Medicine, King Fahad Hospital of the University, Imam Abdulrahman Bin Faisal University, Dammam 32253, Saudi Arabia; Department of Pediatrics, Pediatric Neurology and Metabolic Medicine Unit, Cairo University, Cairo 11628, Egypt; Department of Pediatrics, Pediatric Neurology and Metabolic Medicine Unit, Cairo University, Cairo 11628, Egypt; Broad Center for Mendelian Genomics, Broad Institute of MIT and Harvard, Cambridge, MA 02142, USA; Division of Genetics and Genomics, Boston Children’s Hospital, Harvard Medical School, Boston, MA 02115, USA; Division of Genetics and Genomics, Boston Children’s Hospital, Harvard Medical School, Boston, MA 02115, USA; Howard Hughes Medical Institute, Boston Children’s Hospital, Boston, MA 02115, USA; Department of Neurosciences, University of California San Diego, La Jolla, CA 92093, USA; Rady Children’s Hospital, Rady Children’s Institute for Genomic Medicine, San Diego, CA 92128, USA; Division of Genetics and Genomics, Boston Children’s Hospital, Harvard Medical School, Boston, MA 02115, USA; Howard Hughes Medical Institute, Boston Children’s Hospital, Boston, MA 02115, USA; Department of Pediatrics and Neurology, Harvard Medical School, Boston, MA 02115, USA; Department of Translational Genomics, Genomic Medicine Centre of Excellence, King Faisal Specialist Hospital and Research Center, Riyadh 11564, Saudi Arabia; Lifera Omics, Riyadh 11211, Saudi Arabia; Department of Translational Genomics, Genomic Medicine Centre of Excellence, King Faisal Specialist Hospital and Research Center, Riyadh 11564, Saudi Arabia; Department of Pediatrics, Pediatric Neurology and Metabolic Medicine Unit, Cairo University, Cairo 11628, Egypt; Department of Clinical Genetics, Cambridge University Hospitals NHS Foundation Trust, Cambridge CB2 0QQ, UK; Department of Pediatrics, Pediatric Neurology and Metabolic Medicine Unit, Cairo University, Cairo 11628, Egypt; Department of Neurology, Boston Children’s Hospital, Boston, MA 02115, USA; Rosamund Stone Zander Translational Neuroscience Center, Boston Children’s Hospital, Boston, MA 02115, USA; Max Planck Institute of Biochemistry, Martinsried 82152, Germany; Department of Bioscience, TUM School of Natural Sciences, Technical University of Munich, Garching 85748, Germany; Institut de biologie moléculaire des plantes, CNRS, Université de Strasbourg, Strasbourg 67084, France; IGBMC, Institut de Génétique et de Biologie Moléculaire et Cellulaire, Illkirch 67400, France; CNRS, Centre National de la Recherche Scientifique, UMR 7104, Illkirch 67400, France; INSERM, Institut National de la Santé et de la Recherche Médicale, UMR-S 1258, Illkirch 67400, France; IGBMC UMR 7104-UMR-S 1258, Université de Strasbourg, Illkirch 67400, France; IGBMC, Institut de Génétique et de Biologie Moléculaire et Cellulaire, Illkirch 67400, France; CNRS, Centre National de la Recherche Scientifique, UMR 7104, Illkirch 67400, France; INSERM, Institut National de la Santé et de la Recherche Médicale, UMR-S 1258, Illkirch 67400, France; IGBMC UMR 7104-UMR-S 1258, Université de Strasbourg, Illkirch 67400, France; IGBMC, Institut de Génétique et de Biologie Moléculaire et Cellulaire, Illkirch 67400, France; CNRS, Centre National de la Recherche Scientifique, UMR 7104, Illkirch 67400, France; INSERM, Institut National de la Santé et de la Recherche Médicale, UMR-S 1258, Illkirch 67400, France; IGBMC UMR 7104-UMR-S 1258, Université de Strasbourg, Illkirch 67400, France

**Keywords:** ADAT3, tRNA, deamination, neuronal migration, neurodevelopmental disorders

## Abstract

The ADAT2/ADAT3 (ADAT) complex catalyses the adenosine to inosine modification at the wobble position of eukaryotic tRNAs. Mutations in *ADAT3*, the catalytically inactive subunit of the ADAT2/ADAT3 complex, have been identified in patients presenting with severe neurodevelopmental disorders. Yet, the physiological function of the ADAT2/ADAT3 complex during brain development remains totally unknown.

Here, we investigated the role of the ADAT2/ADAT3 complex in cortical development. First, we report 21 neurodevelopmental disorders patients carrying biallelic variants in *ADAT3*. Second, we used structural, biochemical and enzymatic assays to deeply characterize the impact of those variants on the ADAT2/ADAT3 structure, biochemical properties, enzymatic activity, and tRNAs editing and abundance. Finally, *in vivo* complementation assays were performed to correlate functional deficits with neuronal migration defects in the developing mouse cortex.

Our results showed that maintaining a proper level of ADAT2/ADAT3 catalytic activity is essential for radial migration of projection neurons in the developing mouse cortex. We demonstrated that the identified *ADAT3* variants significantly impaired the abundance and, for some, the activity of the complex, leading to a substantial decrease in inosine 34 levels with direct consequence on tRNAs steady state. We correlated the severity of the migration phenotype with the degree of loss of function caused by the variants.

Altogether, our results highlight the critical role of ADAT2/ADAT3 during cortical development and provide cellular and molecular insights into the pathogenic mechanisms underlying ADAT3-related neurodevelopmental disorders.

## Teaser

Del-Pozo-Rodriguez *et al*. show how mutations in the ADAT complex, which modifies transfer RNA, cause severe neurodevelopmental disorders. They demonstrate that proper ADAT activity is crucial for neuronal migration and reveal connections between genetic variants, enzymatic function, and clinical outcomes.

## Introduction

Cellular homeostasis and growth require protein synthesis to be both efficient to guarantee sufficient production and accurate to prevent the generation of defective or unstable proteins. Efficient and faithful protein translation rely on the activity of transfer RNAs (tRNAs), the adaptor molecules needed to decode genetic information into a peptide sequence. To be fully active, tRNA molecules need to be heavily modified post-transcriptionally. About 30 chemical modifications have been identified at various positions in human tRNAs.^1-3^ On average, a single tRNA carries 13 modifications.^4^ These modifications are catalysed by different classes of tRNA-modifying enzymes and influence tRNA structure, function and stability.^5^ Nucleotides in the anticodon loop are extensively modified.^5^ Those modifications are crucial, as they regulate the tRNA–mRNA interaction to either stabilize cognate Watson–Crick base pairing (position 37) or to facilitate wobble pairing (position 34) to increase the decoding capacity and to prevent frameshift errors.^5^ Thanks to the recent identification of human homologues for many tRNA-modifying enzymes and the wide use of whole exome sequencing, an increasing number of genes encoding for tRNA-modifying enzymes have been linked to human diseases.^3^ Interestingly, neurodevelopmental anomalies are the primary manifestation of variants in genes coding for tRNA-modifying enzymes suggesting a specific sensitivity of the brain during development to perturbations in tRNA modifications.^6^ Although most of those variants have been shown to affect tRNA modification *in vitro*, their direct implication in disease and the underlying pathophysiological mechanisms have only been identified for a few of them.^7-17^

ADAT2/ADAT3 is a heterodimeric enzyme complex that edits adenosine (A) to inosine (I) at the wobble position 34 of tRNAs starting with an A in their anticodon (ANN-tRNAs).^18^ It recognizes specifically tRNAs through the ADAT3 N-terminal domain (ADAT3N), which subsequently rotates to present the bound tRNAs to the ADAT catalytic domain, composed of the C-terminal domain of ADAT3 (ADAT3C) and ADAT2, that carries the catalytic activity.^19-21^ Given the ability of inosine to pair with uracil (U), cytosine (C) or adenosine (A),^22^ the A to I conversion at position 34 provides an extended base pairing capacity to the modified tRNAs and is essential for decoding the C-ending codons, as GNN-tRNAs do not exist in eukaryotic genomes.^23,24^ In accordance, complete deletion or loss of activity of this tRNA modification complex leads to lethality.^18,25-29^ Knocking down the activity of the complex leads to impaired cell cycle progression^30^ and growth retardation^25^ in several species including yeast and humans, possibly due to its role in controlling translational kinetics.^31^

Reflecting a key role of I_34_ modification in brain development, pathogenic variants in *ADAT3* have been identified in patients with neurodevelopmental disorders (NDDs). The same homozygous *ADAT3* variant (NM_138422.4:c.430G>A; p.Val144Met) has been reported in 55 patients from 29 consanguineous families presenting with an autosomal recessive syndromic form of intellectual disability (ID) characterized by developmental delay, moderate to severe ID, speech delay, microcephaly, abnormal brain structure, facial dysmorphism and epilepsy (Supplementary Table 1).^32-38^ In addition to this founder mutation, a homozygous duplication in *ADAT3* (NM_138422.4:c.99_106dupGAGCCCGG; p.Glu36Glyfs*44)^39^ and two compound heterozygous missense *ADAT3* variants in the conserved non-catalytic deaminase domain (NM_138422.4:c.587C>T, p.Ala196Val; c.586_587delinsTT, p.Ala196Leu)^40^ and (NM_138422.4:c.587C>T, p.Ala196Val; c.820C>T p.Gln274*)^17^ have been described in six patients with similar but milder ID syndrome features. The p.Val144Met variant alters the tRNAs A_34_ deaminase activity of the ADAT2/ADAT3 complex^16,21^ possibly through impaired presentation of ADAT2/3 bound tRNAs to the catalytic site without compromising the formation of the complex.^21^ Yet the structural and functional impact of other variants on the ADAT2/3 complex is unknown. Moreover, although I_34_ levels were shown to be decreased in total tRNAs isolated from patients carrying the homozygous p.Val144Met^16^ and compound heterozygous p.Ala196Val; p.Gln274* variants,^17^ our knowledge of the molecular effect of ADAT3 variants on specific ADAT-target tRNAs is very limited and the neurodevelopmental processes that require the proper function of the ADAT2/ADAT3 complex have not been elucidated.

Here, we show that the catalytic activity of the heterodimeric ADAT2-ADAT3 complex is critical to promote the radial migration of projection neurons during corticogenesis. We also expand the molecular spectrum of *ADAT3*-related neurodevelopmental disorders by reporting 21 patients presenting with IDs, structural brain anomalies and global growth retardation carrying the previously identified homozygous p.Val144Met variant or the biallelic p.Ala196Val/p.Ala196Leu variants. Using structural, biochemical, molecular and *in vivo* complementation assays, we showed that, although all the variants act through a loss of function mechanism, they have various effects on complex structure, stability and deamination activity that dictate their ability to restore migration defects upon *Adat3* deficiency. We further drew an exhaustive list of tRNA species affected in disease conditions, providing strong evidence of a causal relationship between variants in *ADAT3*, loss of translationally competent ANN tRNAs and neurodevelopmental disorders.

## Materials and methods

Materials and methods for this manuscript are provided in the Supplementary material. This includes information on whole exome sequencing and patients, cloning and plasmid constructs, production of antibodies, mice, *in utero* electroporation, mouse brain fixation and immunolabelling, primary neuronal culture, cell culture and transfections, RNA extraction, quantitative reverse transcription PCR, western blot, small-scale expression tests of mADAT2/ADAT3, structure of the ADAT complex, enzymatic deamination assays, tRNA-seq, codon enrichment analysis, image acquisition and analysis and statistics.

## Results

### The ADAT2/ADAT3 complex is expressed ubiquitously during cortical development

**Figure 1:**
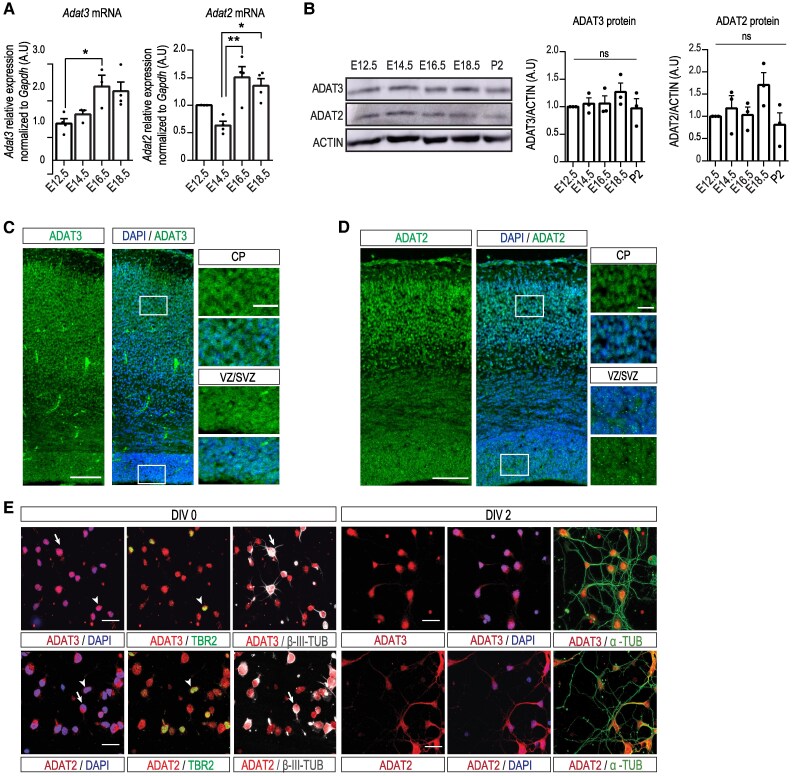
**ADAT2/ADAT3 expression pattern in the mouse embryonic cerebral cortex.** (**A**) Quantitative reverse transcription PCR and (**B**) western blot analysis performed on wild-type mouse cortices showing expression of *Adat3* and *Adat2* transcripts (**A**) and proteins (**B**) levels throughout development from embyronic stage (E) 12.5 to postnatal stage (P) 2 (*n* = 3–5 cortices per stage). Data are presented as means ± standard error of the mean and normalized to E12.5. Significance was calculated by one-way ANOVA (Bonferroni's multiple comparisons test), ns = non-significant; *P < 0.05; **P < 0.01. (**C** and **D**) E18.5 (**C**) and E16.5 (**D**) mouse forebrain coronal sections immunolabelled for (**C**) ADAT3 and (**D**) ADAT2 and counterstained with DAPI (4′,6-diamidino-2-phenylindole) revealing expression of ADAT3 and ADAT2. Close-up views of the white boxed area in CP and VZ/SVZ show localization of ADAT3 and ADAT2 in both progenitors and neurons. CP = cortical plate; SVZ = subventricular zone; VZ = ventricular zone. Scale bars = 100 μm and 50 μm (**C**) or 20 μm (**D**) for magnifications. (**E**) Cortical neurons immunostained for ADAT3, ADAT2, TBR2, α-TUBULIN (α-TUB) and β-III-TUBULIN (β-III-TUB) and counterstained with DAPI at 0 or 2 days *in vitro* (DIV). Arrows point to neurons (cells positive for β-III-TUBULIN). Arrowheads point to intermediate progenitors (cells positive for TBR2). Scale bars = 25 μm.

We first examined the expression pattern of both catalytic and non-catalytic subunits of the ADAT2/ADAT3 heterodimeric complex during mouse cortical development. Although the levels of *Adat3* and *Adat2* mRNA transcripts tend to increase from embryonic Day (E) 12.5 to E18.5 (Fig. 1A), immunoblotting using homemade antibodies for ADAT3 and ADAT2 showed rather stable expression of both proteins (Fig. 1B). Immunolabelling of embryo brain sections revealed localization of ADAT3 and ADAT2 in both progenitors and neurons (Fig. 1C and D, insets). Further analysis of the subcellular localization of each protein on day *in vitro* (DIV) 0 and DIV2 primary cortical neurons showed a diffused expression pattern of the ADAT2/ADAT3 complex (Fig. 1E) in both the cytoplasm and the nucleus.

### ADAT3 regulates the radial migration of projection neurons

**Figure 2:**
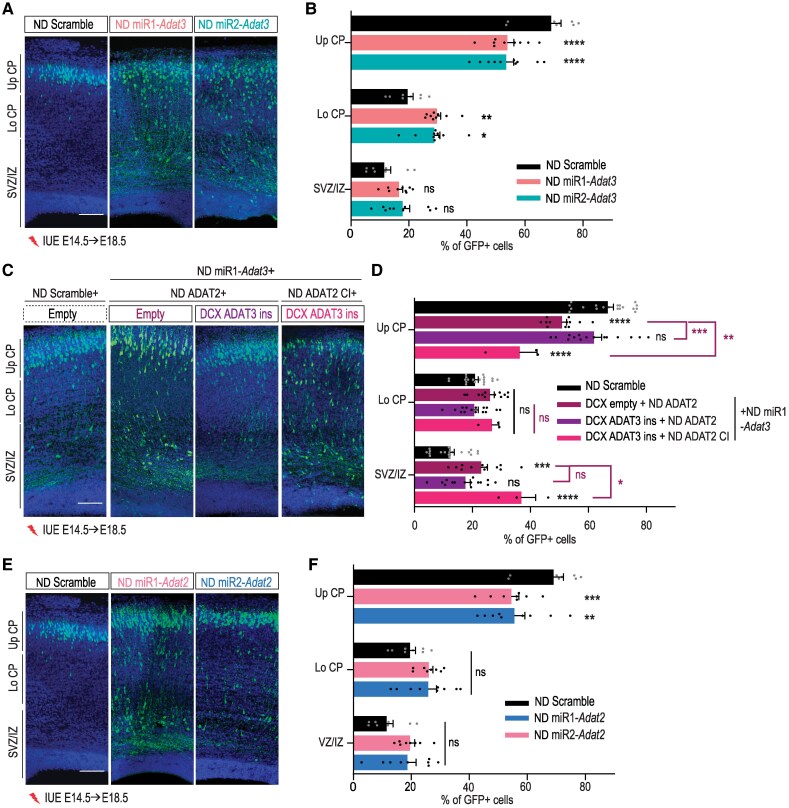
**The role of ADAT3 in migrating neurons depends on its function within the ADAT2/ADAT3 complexes.** (**A**) Coronal sections of embryonic Day (E) 18.5 mouse cortices electroporated at E14.5 with NeuroD (ND) scramble or two distinct ND-*Adat3* microRNAs (miR1 and miR2) together with ND-GFP. (**B**) Percentage [means ± standard error of the mean (SEM)] of the positive electroporated cells (GFP+) in upper cortical plate (Up CP) and lower cortical plate (Lo CP), intermediate (IZ) and subventricular zones (SVZ) showing the faulty migration of *Adat3*-silenced neurons. (**C**) Coronal sections of E18.5 mouse cortices electroporated at E14.5 with ND scramble or ND-*Adat3* miR1 together with empty vector or DCX WT ADAT3 [miR1-insensitive (ins)] and WT or catalytically inactive (CI) ND-ADAT2. (**D**) Percentage (means ± SEM) of the positive electroporated cells in upper (Up CP) and lower (Lo CP) cortical plate, intermediate (IZ) and subventricular zones (SVZ) showing the need of catalytic-active ADAT2 for ADAT3 to rescue the faulty migration of *Adat3*-silenced neurons. (**E**) Coronal sections of E18.5 mouse cortices electroporated at E14.5 with ND scramble or two distinct ND-*Adat2* miRNAs (miR1 and miR2), together with ND-GFP. (**F**) Percentage (means ± SEM) of the positive electroporated cells (GFP+) in Up CP and Lo CP, IZ and SVZ showing the faulty migration of *Adat2*-silenced neurons. Panels **B** and **F** are related; the experiments were performed concurrently using the same ND scramble control group. (**A**, **C** and **E**) GFP-positive electroporated cells are depicted in green. Nuclei are stained with DAPI (4′,6-diamidino-2-phenylindole). Scale bars = 100 μm. (**B**, **D** and **F**) Data were analysed by two-way ANOVA with Bonferroni's multiple comparisons test. Number of embryos analysed: (**B**) NeuroD Scramble, *n* = 8; NeuroD miR1-*Adat3*, *n* = 9; NeuroD miR2*-Adat3*, *n* = 10; (**D**) NeuroD Scramble, *n* = 19; Empty + NeuroD Adat2 + NeuroD miR1-*Adat3, n* = 12; NeuroD Adat2 + DCX Adat3 + NeuroD miR1-*Adat3*, *n* = 15; NeuroD Adat2 C.I + DCX Adat3 + NeuroD miR1-*Adat3*, *n* = 3; (**F**) NeuroD Scramble and NeuroD miR1-*Adat2*, *n* = 8; NeuroD miR2*-Adat2*, *n* = 9; ns = non-significant; *P < 0.05; **P < 0.01; ***P < 0.001; ****P < 0.0001.

To evaluate the function of mouse ADAT3 (mADAT3), we cloned two different microRNAs (miRNA) targeting specifically *Adat3* mRNA (Supplementary Table 2) and confirmed their efficacy by RT-qPCR and immunoblotting (reduction of 85.3% and 96.5% of protein levels for miR1- and miR2-*Adat3*, respectively) (Supplementary Fig. 1A and B). We first assessed the consequences of acute depletion of m*Adat3* on neuronal migration in wild-type (WT) mouse cortices using *in utero* electroporation (IUE) at E14.5 of miRNAs under the control of a ubiquitous CAG promoter together with a NeuroD(ND)-IRES-GFP reporter construct, allowing the expression of GFP (green fluorescent protein) specifically in postmitotic neurons. Four days after IUE, the distribution of GFP+ neurons depleted for *Adat*3 was significantly impaired with a notable reduction of GFP+ neurons reaching the upper cortical plate (up CP) upon acute depletion of *Adat3* (−32.7% and −21.7% for miR1- and miR2-*Adat*3, respectively) (Supplementary Fig. 1C and D). Yet, after birth, most of the *Adat3*-silenced cells showed correct positioning with nearly all cells found in the upper layer of the cortex, indicating a delay in migration rather than a permanent arrest (Supplementary Fig. 1E). As pCAGGS is a ubiquitous promoter, impaired neuronal positioning observed upon pCAGGS-driven *Adat3* deletion might result from defects arising in progenitors, in their neuronal progeny or in both. We performed IUE of plasmids expressing the same miRNAs under the control of the neuronal promoter NeuroD (ND) to induce neuron-specific knockdown. We showed faulty migration of *Adat3*-silenced neurons with a reduction of 21.9% and 22.7% of cells distributed in the upper CP for ND miR1- and ND miR2-*Adat3*, respectively, suggesting that defect in postmitotic neurons largely contributed to the *Adat3*-dependent migration phenotype in a cell autonomous manner (Fig. 2A and B). To further validate the specificity of the migratory phenotype induced by *Adat3* silencing, we tested the ability of WT mADAT3 protein to restore the migration defects. We performed co-electroporation of NeuroD-driven miRNAs together with plasmids expressing miRNA-insensitive mADAT3 under the regulation of a neuronal promoter DCX (Supplementary Fig. 1F). While co-electroporation of WT DCX-ADAT3 alone failed to rescue the impaired distribution of *Adat3*-depleted neurons (Supplementary Fig. 1G and H), *Adat3*-silenced neurons expressing both WT DCX-ADAT3 and NeuroD(ND)-ADAT2 displayed correct positioning within the upper cortical plate (Fig. 2C and D and Supplementary Fig. 1G and H) indicating that the ability of ADAT3 to restore the migration phenotype depends on the stoichiometric expression of ADAT3 and ADAT2 *in vivo*.^16,18^ Of note, ND-ADAT2, alone (Supplementary Fig. 1G and H) or in combination with DCX-ADAT3 (Supplementary Fig. 5B and C) did not induce migration phenotypes while overexpressed under control conditions (sh-Scramble). Altogether, these results demonstrate that ADAT3 is cell autonomously required for the proper migration of projection neurons and suggest a contribution of tRNAs modification to radial migration.

### The catalytic activity of the ADAT2/ADAT3 complex is required for proper neuronal migration

To assess whether the migratory function of ADAT3 depends on its function within the heterodimeric ADAT2/ADAT3 enzymatic complex, we next explored the effect of silencing the catalytically active partner, ADAT2, on neuronal migration. We performed acute depletion of *Adat2* specifically in neurons by IUE of NeuroD-driven *Adat*2-miRNAs in WT cortices at E14.5. The ability of miRNAs to efficiently and specifically target *Adat2* was tested by qPCR and immunoblotting (reduction of 83% of protein levels for both miR1- and miR2-*Adat2*) (Supplementary Fig. 1A and B and Supplementary Table 2). Consistent with a critical role of the complex in the regulation of radial migration, the migration defects observed after depletion of *Adat2* were comparable to those observed after silencing of *Adat3* (−21%, −19.5% of cells reaching the upper cortical plate in ND miR1- and ND miR2-*Adat2*, respectively) (Fig. 2E and F). We next addressed whether the ADAT2/ADAT3 complex controls neuronal migration through its catalytic activity and tested for rescue of the phenotype induced by the loss of *Adat3* by expressing WT DCX-ADAT3 and a catalytically inactive form of ADAT2 (ND-ADAT2 CI, Supplementary Fig. 1I).^18,21,41^ In accordance with the need of enzymatic activity, co-expression of ADAT3 with WT ADAT2 but not with catalytic inactive ADAT2 rescued the impaired positioning of *Adat3*-depleted cells (Fig. 2C and D). Altogether, these results demonstrate the catalytic activity of the ADAT2/ADAT3 complex is required to exert its function in migrating projection neurons.

### Identification of novel patients with ADAT3 variants

**Figure 3:**
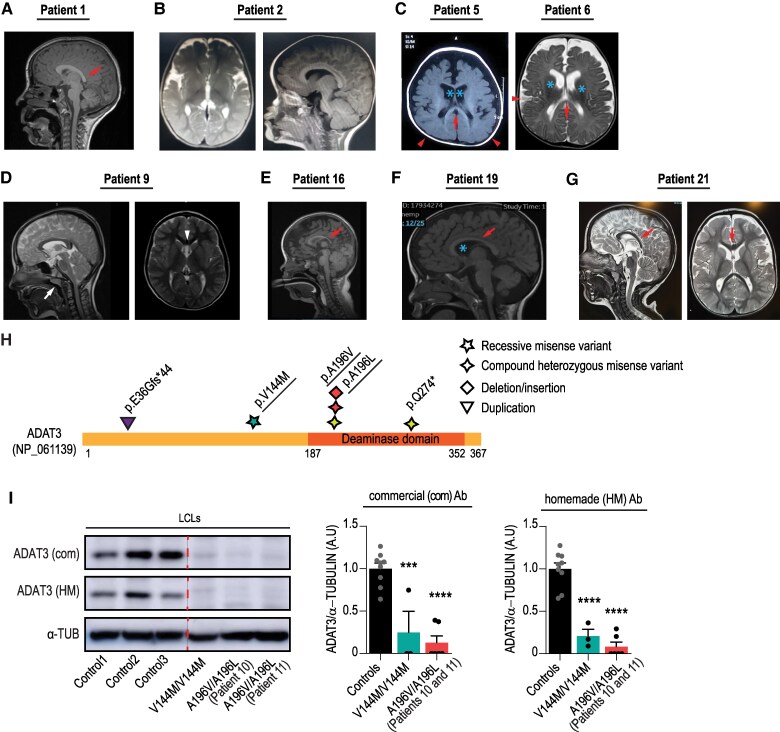
**Clinical features of patients with *ADAT3* variants.** (**A**–**F**) Axial and/or sagittal T1- and T2-weighted brain MRI images of (**A**) Patient 1, (**B**) Patient 2, (**C**) Patients 5 and 6, (**D**) Patient 9, (**E**) Patient 16, (**F**) Patient 19 and (**G**) Patient 21. The red arrows point to thin corpus callosum, red arrowhead indicates the simplified gyral pattern of the cortex. Asterisks show enlarged ventricles. White arrow and arrowhead indicate, respectively, adenoid hypertrophy and cavum septum pellucidum. (**H**) Schematic representation of human ADAT3 protein indicating positions of all variants identified so far. Variants depicted in the same colour were found in the same patient. *In vivo* functional tests have been performed for the variants that are underlined. (**I**) Western blot analysis of p.V144M/p.V144M and p.A196V/p.A196L (Patients 10 and 11) patient cells revealing reduced ADAT3 protein levels in comparison to controls [controls, *n* = 9; V144M/V144M, *n* = 3 and A196V/A196L, *n* = 6 (three of each patient)]. α-Tubulin (α-TUB) is used as a protein loading control. Both commercial (com) and homemade (HM) antibodies have been used to detect ADAT3 proteins. Red dashed line indicates where the membrane was cut. One-way ANOVA, Bonferroni's multiple comparisons test. ***P < 0.001; ****P < 0.0001. LCL = lymphoblastoid cell lines.

Through the GeneMatcher^42^ and Matchmaker Exchange^43,44^ platforms, we identified 21 individuals from 18 unrelated families carrying biallelic variants in *ADAT3*, presenting with IDs and brain malformation (Supplementary Table 1). These individuals included 12 males and 9 females with ages ranging from 9 months to 16 years. Of note, Patient 1 has been previously reported in a cohort of 50 probands with cerebral palsy.^33^ Patients with Middle East origin (19/21) (Patients 1–9 and 12–21) carry the homozygous p.Val144Met (p.V144M) variant (NM_138422: c.430G>A), the most common cause of autosomal recessive ID in Arabia, whereas previously published siblings with of European descent (2/21) (Patients 10 and 11)^40^ have the compound heterozygous variant p.Ala196Leu(p.A196L)/p.Ala196Val (p.A196V) (NM_138422: c.587C>T; c586_587delinsTT) (Supplementary Fig. 2A–Q). In accordance with previous reports,^17,32-37^  ^,39^  ^,40^ commonly observed clinical features included global developmental delay (20/20), ID (19/19), muscle tone defects (16/20), microcephaly (11/19) and epilepsy (7/21) (Supplementary Tables 1, 3 and Supplementary material, note 1). All patients presented with motor delay and language deficit ranging from severely impaired speech to non-verbal. MRI images were available for 16 patients (Patients 1, 2, 5–7, 9, 10, 12–19 and 21). While Patients 7, 10, 12–15, 17 and 18 showed a normal brain structure, other patients displayed variable brain structural anomalies including dysplastic appearance of the corpus callosum (Patients 1, 5, 6, 16, 19 and 21), microcephaly (Patients 2, 5, 16 and 21), abnormal gyrification (Patients 5 and 6), enlarged ventricles (Patients 5, 6, 16 and 19), cavum septum pellucidum (Patient 9) and nearly absent myelination (Patient 5) (Fig. 3A–G). Overall, we expanded the clinical spectrum of *ADAT3*-related neurodevelopmental disorders by presenting 21 patients displaying severe neurodevelopmental delay associated with heterogenous brain anomalies (Fig. 3H, Supplementary Tables 1, 3 and Supplementary material, note 1). To further interrogate the molecular effect of the *ADAT3* variants, we analysed the level of expression of ADAT3 in patients’ samples when available. We compared hADAT3 protein levels in lymphoblastoid cell lines (LCLs) homozygous for the p.V144M variant (affected patient depicted in Supplementary Fig. 2R—Fam1 in Alazami *et al*.^32^) and p.A196V/p.A196L (Patients 10 and 11) depicted in Supplementary Fig. 2S to control lymphoblasts generated from sex- and age-matched healthy individuals. Though the levels of *ADAT3* transcripts remained stable (Supplementary Fig. 2T), immunoblotting with two different antibodies revealed that both the ADAT3 p.V144M and p.A196V/p.A196L variant lead to a severe but not total depletion of ADAT3 protein levels (Fig. 3I), in line with the total loss of ADAT3 being incompatible with life.^18,25-29^

### Variants in ADAT3 affect the stability, structure and enzymatic activity of the ADAT2/ADAT3 complex

**Figure 4:**
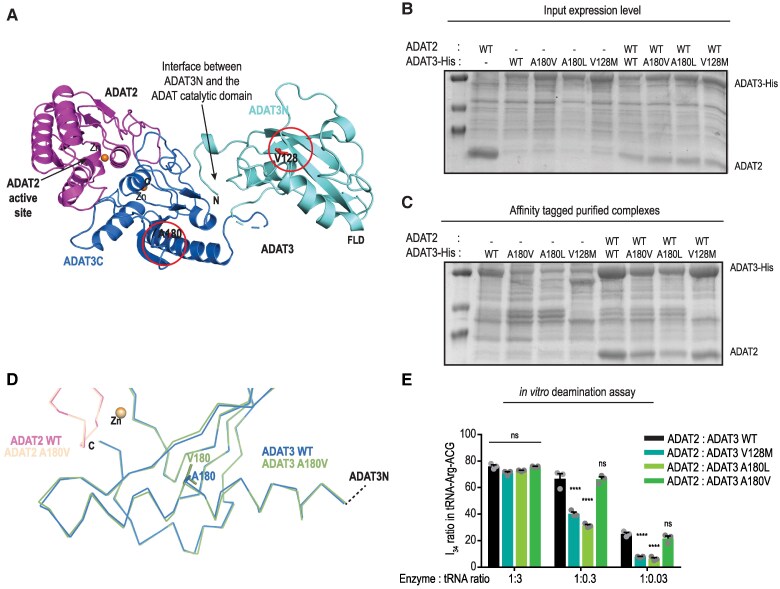
**The V128M and A180V/L ADAT3 mutants affect ADAT2/ADAT3 stability, structure and deamination activity.** (**A**) Ribbon representation of the crystallographic structure of the wild-type (WT) mouse ADAT complex (PDB entry 7nz8). The catalytic domain of ADAT is composed of ADAT2 (magenta) and the C-terminal domain of ADAT3 (blue; ADAT3C). The N-terminal domain of ADAT3 (cyan; ADAT3N) is key to recognizing tRNAs through its ferredoxin-like domain (FLD) and to rotate with respect to the ADAT catalytic domain to position the tRNA anticodon loop within the ADAT2 active site. The two residues (V128 and A180), shown in red and that are found mutated in patients, are displayed. These are located in different regions of the ADAT complex. (**B**) SDS-PAGE (sodium dodecyl-sulfate polyacrylamide gel electrophoresis) analysis of expression levels in *E. coli* of untagged ADAT2 WT and His-tagged ADAT3 (WT and A180V, A180L, V128M mutants) constructs, either alone or in combination. Expression levels are similar for all constructs used. (**C**) SDS-PAGE analysis of His-tag affinity-purified samples of **B**. In the absence of ADAT2, the ADAT3 mutant constructs show a significant decrease in solubility compared with WT. Co-expression of ADAT2 with these constructs restore solubility upon formation of the ADAT2/ADAT3 complex albeit to different extents. (**D**) Close-up view and comparative structural analysis of the region of the ADAT complex harbouring A180 in the ADAT2/ADAT3 and ADAT2/ADAT3-A180V complex structures. The A180V mutation induces local changes in the main chain neighbouring secondary structure elements, including in the central β-sheet organizing the ADAT3 C-terminal domain. (**E**) Deamination assays for mouse ADAT3 WT, A180V, A180L and V128M in complex with ADAT2. Whereas the WT and A180V complexes have similar activities, the A180L and V128M complexes show a similar decrease in activity. Data (means ± standard error of the mean) from three different experiments per condition were analysed by two-way ANOVA, with Dunnett's multiple comparison test. ns = non-significant; ****P < 0.0001.

We next evaluated the impact of the identified variants on the remaining ADAT2/ADAT3 complex. We first mapped the variants using the crystal structure of the mouse WT ADAT2/ADAT3 complex.^21^ The ADAT3 V128 residue (corresponding to the V144 residue in humans, Supplementary Fig. 3A) is part of a large hydrophobic core located in the middle of the N-terminal domain of ADAT3 (ADAT3N),^21^ while the ADAT3 A180 residue (corresponding to the A196 residue in humans), is buried within the ADAT3 C-terminal domain (ADAT3C, Fig. 4A). Despite their different 3D locations, the p.V144M and p.A196V/L variants cause similar clinical phenotypes raising the question of the impact of these mutations on ADAT3 and ADAT2/ADAT3 complex solubility, structure and activity.

Bacterial expression of the WT and mutant mouse ADAT3 constructs alone showed that, despite similar expression levels (Fig. 4B), all three mutants significantly affect the solubility of ADAT3 (Fig. 4C). However, co-expression of these constructs with ADAT2 partially restored ADAT3 solubility upon formation of the ADAT2/ADAT3 complex. Co-expressed with ADAT2, the V128M ADAT3 construct was almost as soluble as the WT construct, whereas the A180V and A180L constructs were less soluble, albeit the A180V being slightly more soluble than the A180L construct (Fig. 4C). Interestingly, as assessed during the purification process, once formed and soluble, the mutant complexes do not show aggregation properties and can be used for further biochemical assays and structural analyses. We therefore sought to characterize the structural effect of the A180V/L mutations as was done previously for the variant at the V128 residue.^21^ In our initial study, crystals were obtained for the ADAT2/ADAT3-p.V128M complex but those did not diffract sufficiently. However, the structure of the ADAT2/ADAT3-V128L complex showed that the V128L mutation perturbs the ADAT3N region involved in the interaction with the ADAT catalytic domain, suggesting a partially impaired presentation of the bound tRNA anticodon loop to the ADAT2 active site. These defects should be exacerbated in the case of the V128M mutant.^21^ Crystallization assay of the ADAT2/ADAT3-A180V and ADAT2/ADAT3-A180L complexes were successful for the former complex, while the latter failed to crystallize. Following structure determination at 2.9 Å resolution (Supplementary Table 4), comparison with the structure of the WT ADAT2/ADAT3 complex revealed that, unlike the p.V128L variant that mostly affects the ADAT3N, the p.A180V substitution causes small local perturbations in the structure of the ADAT3 C-terminal domain (Fig. 4D). We anticipate in this case also that this effect is exacerbated with the A180L mutant due to the bulkier character of the leucine residue compared with the valine residue. The structural effects caused by the mutation of the A180 residue are more difficult to predict than those of the V128 residue.^21^ Indeed, this residue is relatively far away from ADAT2 and its active site, and it is unlikely that mutation of this residue directly affects the catalytic mechanism. On the other hand, A180 is located in the long α-helix that immediately follows the ADAT3N domain (Fig. 4A). Its mutation into leucine could affect the proper folding and positioning of this helix but also those of the central ADAT3 C-terminal domain β-sheet that is also involved in the interaction with ADAT3N. Therefore, our structural analysis suggested that the A180L mutation probably also affects the interaction between the ADAT3N domain and the ADAT catalytic domain, thereby hampering the correct presentation of the tRNA to the ADAT catalytic domain and, consequently, the deaminase activity.

To ascertain the effect of the variants on the enzymatic activity of the ADAT2/ADAT3 complex, we performed sequencing of an *in vitro*-transcribed cognate tRNA, tRNA Arg(ACG), after incubation with different amounts of purified recombinant WT or mutant ADAT2/ADAT3 complexes.^21^ As inosine is read as a ‘G’ by reverse transcriptase,^28^ we sought the percentage of G_34_ as a proxy of A_34_ to I_34_ editing. While the p.V128M ADAT3 exhibited a significantly impaired enzymatic activity (−68% of A_34_ to I_34_ with the lowest concentration of the ADAT2/ADAT3-V128M complex) as expected,^16,21^ the ADAT2/ADAT3-A180V heterodimer retained comparable activity to the WT complex and the ADAT2/ADAT3-A180L displayed a severely diminished deamination capacity (−76% compared with the WT complex at the lowest concentration) (Fig. 4E and Supplementary Fig. 3B). Notably, the two variants found in the Patients 10 and 11 (p.A180V/L; corresponding to p.A196V/L in humans) impair ADAT2/ADAT3 deamination activity differently, although they affect the solubility of the ADAT2/ADAT3 complex similarly (Fig. 4C). Along the same lines, the p.V128M ADAT3 variant strongly affected the enzymatic activity of the complex despite the lack of any effect on its solubility (Fig. 4C and E). Overall, these data strongly suggest that the level of deamination activity does not directly correlate to the level of solubility but rather indicate that the faulty deamination activity of the mutant complexes might mostly stem from structural perturbations (Table 1).

**Table 1:** Summary table showing that the loss of enzymatic activity correlates with structural alterations rather than with impaired solubility

Compared with wild-type	V128M	A180V	A180L
Solubility	=	↓↓	↓↓↓
Structural perturbations	Strong^a^	Mild	Strong^b^
Activity	↓↓	=	↓↓↓

^a^Prediction based on the structure of ADAT2/ADAT3-V128L.

^b^Prediction based on the structure of ADAT2/ADAT3-A180V.

### Selective loss of I_34_ modification and reduced steady state of cognate tRNAs in patient-derived cells

**Figure 5:**
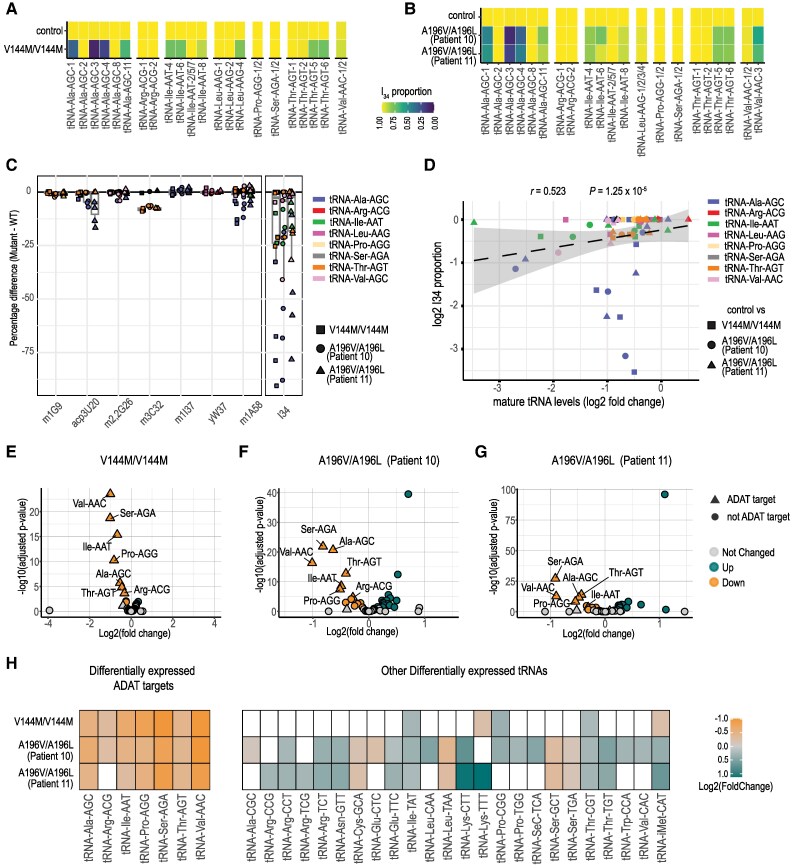
**Deamination and abundance of ADAT2/ADAT3 target tRNAs are decreased in patient cells.** (**A** and **B**) Heat map showing I_34_ levels in ADAT target tRNA isodecoders in LCLs (lymphoblastoid cell lines) derived from (**A**) p.V144M/ p.V144M and (**B**) p.A196V/p.A196L patients compared with controls. [Controls, *n* = 2; V144M/V144M, *n* = 2; A196V/A196L (Patient 10) *n* = 3; A196V/A196L (Patient 11) *n* = 3]. (**C**) Graph showing the percentage change in ADAT target tRNAs of all other seven modifications (m^1^G9, acp^3^U20, m^2^_2_G26, m^3^C32, m^1^I37, yW37, m^1^A58) that can be detected by mim-tRNA seq. The change in I_34_ levels is depicted as a side view for comparison. tRNA isoacceptors from comparison of control LCLs to LCLs derived from V144M/V144M, A196V/A196L (Patient 10) and A196V/A196L (Patient 11) are depicted with squares, circles and triangles, respectively. Isoacceptors are coloured as indicated. (**D**) Graph showing the correlation between the change in I_34_ proportion and the change in mature tRNA levels in log_2_ scales. Black dashed line is the trend line and standard deviation is shown with a grey zone. tRNA isodecoders from comparison of control LCLs to LCLs derived from V144M/V144M, A196V/A196L (Patient 10) and A196V/A196L (Patient 11) are depicted with squares, circles and triangles, respectively. Isodecoders are coloured as indicated. (**E**–**G**) Volcano plot showing the negative log_10_ adjusted *P*-value (*P*-adj) of all tRNAs pooled at the anticodon level against their log_2_ fold change (log_2_FC) in LCLs derived from (**E**) p.V144M/p.V144M (*n* = 2), (**F**) p.A196V/p.A196L (Patient 10, *n* = 3) and (**G**) p.A196V/p.A196L (Patient 11, *n* = 3) compared with controls (controls, *n* = 2). Triangles and circles show ADAT targets and non-target tRNAs, respectively. Green, orange and grey represent upregulated, downregulated and unchanged tRNAs, respectively, based on DESeq2 *P*-adj < 0.05. (**H**) Heat map showing log2 DESeq2 fold change of differentially regulated ADAT target tRNAs and non-target tRNAs summed by anticodon. White boxes show non-significant ones.

Given that ADAT3 variants have various effects on the structure, stability and enzymatic activity of the ADAT2/ADAT3 complex, we next investigated whether the impaired ADAT2/ADAT3 function affects the A_34_ to I_34_ tRNA editing. We took advantage of the recently developed mim-tRNA-seq method that allows robust quantification of individual tRNA species as well as determination of the presence and stoichiometry of eight tRNA modifications, including inosine.^45,46^ In our dataset, >72% of the reads were unique and mapped at 95% on average to nuclear-encoded tRNAs (Supplementary Fig. 4A and B). More than 67% of the uniquely mapped tRNAs were full length tRNAs of which >97% contained the 3′CCA tail indicating that they were mature, translationally competent tRNAs (Supplementary Fig. 4C and D). We first compared the I_34_ proportion in patient and control LCLs at the level of isodecoders, defined as tRNA transcripts sharing the same anticodon but differing elsewhere in their sequence. The isodecoders of the eight ANN tRNA families (Ala-AGC, Arg-ACG, Ile-AAU, Leu-AAG, Pro-AGG, Ser-AGA, Thr-AGU and Val-AAC) are fully deaminated in control conditions, as previously described in other cellular contexts^18,46,47^ (Fig. 5A and B and Supplementary Table 5). However, we observed a consistent deamination defect in both p.V144M/p.V144M and p.A196V/p.A196L patient cells. While isodecoders belonging to the tRNA-Arg-ACG, tRNA-Pro-AGG and tRNA-Ser-AGA families were not affected, the other tRNAs showed a substantial decrease in I_34_ proportion, the tRNA-Ala-AGC family being the most affected with four isodecoders out of the six detected showing a decreased A_34_ to I_34_ editing (mean of 43%, 13%, 31% and 79% of I_34_ for, respectively, Ala-AGC-1 Ala-AGC-3, Ala-AGC-4 and Ala-AGC-11 in the three mutant cell lines) (Fig. 5A and B and Supplementary Table 5). As a result, when grouped by anticodon pools (see the ‘Material and methods’ section in the Supplementary material), we observed a 16%–25% decrease of deamination for Ala-AGC in both mutant conditions compared with the control, while we did not detect any changes in the level of I_34_ for the other seven ANN anticodon families (Supplementary Fig. 4E and F and Supplementary Table 5). Of note, among the other modifications that could be identified by mim-tRNA-seq (m^1^A, m^1^G, m^2^_2_G, m^3^C, yW, acp^3^U, I, m^1^I, m^3^C_32_ and m^1^A_58_), some isodecoders showed a very slight decrease in the patient-derived cell lines compared with controls, confirming the specificity of the I_34_ perturbation and the lack of interdependence of I_34_ with these other detected modifications (Fig. 5C).

We next used mim-tRNA-seq data to compare the abundance of mature tRNAs in control and ADAT3 mutant LCLs. Among the 379 cytoplasmic tRNAs isodecoders for which we reached a single-transcript resolution (90% of the predicted cytoplasmic tRNAs), 19%, 25% and 28% were differentially expressed in p.V144M/p.V144M and in the two p.A196V/p.A196L (Patients 10 and 11) mutant cells compared with the control, respectively (adjusted *P* ≤ 0.05) (Supplementary Fig. 4G–I and Supplementary Table 5). Interestingly, up to 37.5% of the differentially expressed tRNAs are ADAT2/ADAT3 substrates with 57% of all ANN isodecoders being deregulated versus 30% of non-INN tRNAs transcripts (Supplementary Table 5). These data were highly reproducible among replicates as shown in the principal component analysis (Supplementary Fig. 4J). Strikingly, we observed a significant inverse correlation (Spearman's correlation coefficient *r* = 0.523, *P* = 1.25 × 10^−5^) between the decrease in cellular abundance of mature tRNA isodecoders and their level of deamination (I_34_) (Fig. 5D), indicating that the stability of ANN isodecoders is very sensitive to the loss of I_34_. We then aggregated all tRNAs by their anticodon and reperformed a differential expression analysis. We showed that, although some anticodon families that are not targets of the ADAT2/ADAT3 complex were deregulated (Fig. 5E–G, circle points and Supplementary Fig. 4K), six of the eight tRNAs anticodon families, that showed a decreased expression of up to 2-fold in all three mutant cells lines relative to control cells, were ANN tRNAs (Fig. 5H). Of note, the two other non-ANN tRNA commonly deregulated were upregulated (Thr-CGT and lle-TAT).

Overall, these results indicate that the combined decreased expression of the ADAT2/ADAT3 complex and diminished activity of the remaining complexes observed in ADAT3 mutant conditions significantly change the cellular pools of ANN tRNAs, that likely stem from both an excessive proportion of non-translationally competent A_34_ tRNAs and selective degradation of unstable A_34_ tRNA isodecoders.

### Missense variants in *ADAT3* impair neuronal migration through a loss-of-function mechanism

**Figure 6:**
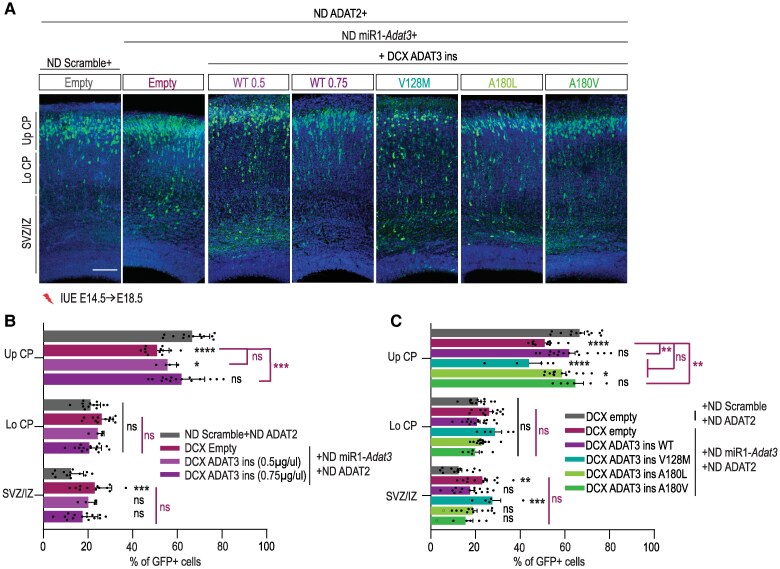
**Missense variants in *Adat3* impair neuronal migration**. (**A**) Coronal sections of embryonic Day (E)18.5 mouse cortices electroporated at E14.5 with NeuroD (ND) ADAT2 and ND-GFP together with either ND scramble or ND *Adat3* miRNAs in combination with DCX Empty; DCX ADAT3 [miR1-insensitive (ins)] at two different concentrations (0.5 or 0.75 µg/µl) or various variants (at 0.75 µg/µl). GFP-positive electroporated cells are depicted in green. Nuclei are stained with DAPI (4′,6-diamidino-2-phenilindole). Scale bar = 100 μm. (**B** and **C**) Analysis of percentage (means ± standard error of the mean) of electroporated cells in upper (Up CP) and lower (Lo CP) cortical plate, intermediate (IZ) and subventricular zone (SVZ) showing a dose-dependent rescue of migration with wild-type proteins and absence of rescue with most of the variants. Data were analysed by two-way ANOVA (Tukey's multiple comparison test). Number of embryos analysed: NeuroD Scramble, *n* = 13; NeuroD miR1-*Adat3* + Empty*, n* = 12; NeuroD miR1-*Adat3* + DCX WT (0.5 μg/μl), *n* = 4; NeuroD miR1-*Adat3* + DCX WT (0.75 μg/μl), *n* = 15; NeuroD miR1-*Adat3* + DCX V128M, *n* = 5; NeuroD miR1-*Adat3* + DCX A180L, *n* = 13; NeuroD miR1-*Adat3* + DCX A180V, *n* = 6; ns = non-significant; *P < 0.05; **P < 0.01; ***P < 0.001; ****P < 0.0001.

To assess the functional consequences of *ADAT3* variants and to ascertain the predicted loss of function mechanism, we evaluated the ability of the variants to restore the migration phenotype induced by the neuronal depletion of *Adat3*. As we observed a decrease in protein levels in both p.V144M/p.V144M and p.A196V/p.A196L ADAT3 LCLs (Fig. 3M), we first tested whether the rescue of the migration phenotype observed upon m*Adat3* deletion depends on ADAT3 dosage by IUE of ND-miR1-*Adat3* together with an increasing amount of DCX-mADAT3. Whereas *Adat3*-silenced neurons expressing 1 unit (0.75 µg/µl) of mADAT3 are correctly distributed in the upper cortical plate 4 days after IUE (Fig. 2C and D), expression of two-thirds of a unit (0.5 µg/µl) of mADAT3 only partially rescued the faulty migration (Fig. 6A and B), suggesting that ADAT3 controls neuronal migration in a dose-dependent manner. Next, to undoubtedly confirm the loss of catalytic activity of the remaining complexes, we performed complementation assays by expressing p.V128M, p.A180V and p.A180L DCX-mADAT3 variants (corresponding to human p.V144M, p.A196V, p.A196L variants, respectively) together with WT ND-mADAT2 in *Adat3*-silenced neurons. When co-expressed with ADAT2, all variants showed expression similar to the expression of the WT protein and their expression in control conditions (Scramble miRNA) did not induce any migration phenotype (Supplementary Fig. 5). Interestingly, the p.A180V variant that does not affect the deamination activity of the ADAT complex as demonstrated *in vitro* (Fig. 4E) restored the migration defects as efficiently as the ADAT3 WT construct (Fig. 6A and C). On the contrary, the p.A180L and p.V128M variants that strongly impaired the activity of the ADAT2/ADAT3 complex (Fig. 4E), failed to rescue the migration phenotype observed upon *Adat3* depletion (Fig. 6A and C). Altogether, these results demonstrate that missense hADAT3 variants impede the radial migration of projection neurons through either loss of expression and/or catalytic activity.

## Discussion

Our findings highlight a critical role of the heterodimeric enzyme complexes, ADAT2/ADAT3, in the regulation of radial migration of projection neurons. We provide several lines of evidence which suggest that the catalytic activity of the ADAT2/ADAT3 complex is required for proper neuronal migration. First, we demonstrated that silencing of both the catalytic (ADAT2) and non-catalytic (ADAT3) subunits of the complex impaired neuronal migration to a similar extent (Fig. 2). Second, the co-expression of ADAT3 together with ADAT2 is required to abolish the phenotype induced by the loss of *Adat3* (Supplementary Fig. 1G and H), suggesting that co-abundance of ADAT2 is likely necessary to stabilize ADAT3 *in vivo*. This result correlates with *in vitro* findings showing that ADAT3 tends to self-associate and aggregate when not assembled with ADAT2.^16^ In addition, co-expression of the catalytic-inactive form of ADAT2 and ADAT3 is unable to rescue the *Adat3*-induced migratory phenotype (Fig. 2E and F). Third, while the ADAT2/ADAT3 complex bearing the p.A196V (A180V mADAT3) variant, that retained a similar enzymatic activity than the WT ADAT complex, restored the faulty migration observed upon *Adat3* depletion; the ADAT2/ADAT3 complexes bearing the p.V144M (V128M mADAT3) and the p.A196L (A180L mADAT3) variants, respectively, which exhibit reduced enzymatic activity lost their ability to rescue the migration phenotype (Figs 4E and 6). Fourth, combined decreased expression and reduced catalytic activity of the ADAT2/ADAT3 complex in patient-derived cell lines impaired the A to I conversion at the wobble position of tRNAs, to an extent that is compatible with life but that likely causes detrimental brain phenotypes (Figs 5 and 6).

We examined the pathological mechanisms associated with the ADAT2/ADAT3 complex-related NDD at the genetic, structural/biochemical and molecular levels. All results converged towards a loss of function mechanism. At the genetic level, we expanded the clinical spectrum of ADAT-related NDDs by reporting 19 new patients from 17 different families carrying the previously identified^32-37^ p.V144M/pV144M variant and by providing clinical updates for two previously reported patients^40^ carrying the biallelic p.Ala196Val/p.Ala196Leu variant. Clinical presentation and MRI of the newly identified individuals matched with the clinical features observed in patients with ADAT-related NDDs.^17,32-37,39,40^ Interestingly, variants in the *ADAT3* gene identified in patients with neurodevelopmental disorders were only found at the biallelic state (Fig. 3 and Supplementary Table 1). Consistently, human *ADAT3* and *ADAT2* genes tolerate loss-of-function variants with many loss-of-function heterozygous variants reported in the gnomAD general population (Genome Aggregation Database, v.4.0.0), suggesting that cortical development can sustain *ADAT3* hemizygosity. Of note, no biallelic null variant has been found in *ADAT2* yet. Corroborating the human findings, we showed that full knock-out of *Adat3* is lethal in mice (data not shown), and that expression of WT mADAT3 restored the neurodevelopmental phenotype in a dose-dependent manner (Fig. 6A and B). Altogether, these findings indicate that a minimal level of complex activity is required to ensure proper mammalian neuronal development.

We provide further insights into the structural basis of the ADAT3-related NDD. Although the V128 (V144 in humans) and A180 (A196 in humans) residues lie in different structural domains, the N-terminal (ADAT3N)^21^ and C-terminal domains, respectively, the V128M and A180L variants are both suggested to affect the presentation of the tRNAs anticodon loop to the active catalytic site of the complex, yet through distinct structural perturbation. Based on the crystal structure of the ADAT2/V128L-ADAT3 mouse complex, we previously predicted that the V128M variant hinders the proper positioning of the ADAT3N terminal domain to correctly present the anticodon loop to ADAT2, likely through perturbation of the interaction between ADAT3N and the ADAT2/ADAT3 catalytic domain.^21^ Here, the crystal structure of the ADAT2/A180V-ADAT3 mutant complex did not reveal any defect in the ADAT3N domain, but rather local perturbations in the α-helix, where the A180 residue is located, and in the ADAT3 C-terminal domain β-sheet, both located close to the interaction surface with ADAT3N. We anticipated that the A180L substitution aggravates these local perturbations and alters the interaction between the ADAT3N and the ADAT2/ADAT3 catalytic domains and therefore, the correct presentation of the tRNAs to ADAT2. This structurally predicted loss of enzymatic activity of the ADAT2/V128M-ADAT3 and ADAT2/A180V-ADAT3 but not of ADAT2/A180L-ADAT3 is supported by: (i) a severe decrease of the tRNAs deamination activity of the V128M and A180L complexes compared with the WT and A180V complexes *in vitro* (Fig. 4E); and (ii) the lack of rescue of the migration phenotype with the two variants showing an impaired enzymatic activity (Fig. 6). Intriguingly, we also showed impaired solubility of the variants (Fig. 4C). However, the structural perturbations observed in the mutant complexes (V128M/L > A180L >> A180V > WT) correlate better with the defects in enzymatic activity (V128M/L; A180L > WT; A180V = WT) than the defects in solubility (A180L > A180V >> V128M/L = WT) (Table 1). Altogether, these results suggest that, although one cannot exclude that the variants preclude the formation of the complexes *in vivo*, the structural alterations are likely one of the major determinants of the loss of deamination activity of the mutant complexes and might greatly contribute to the development of NDDs.

At the molecular level, we provide the first exhaustive quantitative analysis of the impact of *ADAT3* variants on both deamination and expression of ADAT2/ADAT3 target tRNAs. Analysis of the proportion of wobble inosine (I_34_) in ANN isodecoders in cells derived from patients carrying either the p.V144M/p.V144M or p.A196V/p.A196L variants revealed a striking decrease of A_34_ to I_34_ editing in about 45% of the INN isodecoders (Fig. 5). This extends the initial observations showing impaired deamination on a few tRNA species in patients’ cells expressing the p.V144M/p.V144M variant.^16^ Of note, in contrast to ADAT2-depleted human HEK293T cells, where all the ADAT substrate tRNA families showed severe defects in deamination,^27^ in ADAT3 patients’ cells, only the tRNA-Ala family is affected (Supplementary Fig. 4E and F), suggesting a cell-specific effect of ADAT perturbation. Corroborating this hypothesis, it has been shown that proliferating versus differentiating cells translate the codons read by I_34_ tRNAs with different efficiency.^48^ In addition, tRNA-Ala anticodon is one of the only enriched families in the embryonic brain,^49^ suggesting further potential brain sensitivity to ADAT impairment.

Interestingly, the isodecoders of a tRNA family are not affected to the same extent (ranging from 100% I_34_ to 11% for the more severely affected isodecoders). One possible explanation for this differential effect on isodecoders within a same tRNA family is that the mutations in the mammalian ADAT2/ADAT3 complex selectively affect the binding and/or accommodation of specific tRNAs to its catalytic site. Interestingly, the structure of the *Trypanosoma brucei* ADAT2/ADAT3 complex bound to a full length tRNA showed that the eukaryotic ADAT2/ADAT3 complex selects and correctly positions the tRNA substrate within the catalytic site through sequence-independent interactions.^19^ Additional biochemical analysis using chimeric tRNAs or fragments of tRNAs confirm that tRNA structural features are key for tRNA recognition by human ADAT and suggest that the mode of recognition of tRNA by the ADAT2/ADAT3 complex varies between different tRNAs.^50^ A non-exclusive alternative is that I_34_ specifically stabilizes some tRNAs more than others. In such cases, some tRNAs would be found as both I_34_ and A_34_, while others would be found only as I_34_, the A_34_ species being degraded. This is supported by the fact that: (i) our analysis of tRNA abundance showed that most tRNAs commonly downregulated in all three patient samples were ADAT targets (Fig. 5); and that (ii) the extent of I_34_ loss in target tRNA molecules negatively correlate with the degree of downregulation (Fig. 5D). How the lack of I_34_ modification could affect the stability of specific tRNAs remains unknown. While some specific structural determinants might be at play, the often very limited sequence changes between isodecoders suggest that additional mechanisms might be involved. Although none of the modifications detected by mim-tRNAseq, including m^1^A at position 58 of the tRNA-Val-AAC, Thr-AGU and Pro-AGG,^16,17^ are severely impaired in the disease context, one cannot exclude that other I_34_-dependent tRNA modifications that are known to stabilize tRNAs (i.e. methylation)^51,52^ could account for the decreased steady-state level of some but not other ADAT2/ADAT3 cognate tRNAs. For example, the varying sensitivity of the different Ala isodecoders (Fig. 5D) may arise from specific modifications that preferentially stabilize certain tRNA-Ala species. Considerable evidence is in favour of crosstalk between I_34_ and the modification of other bases. First, we previously showed that the recombinant *Escherichia coli* TadA enzyme co-purifies exclusively with its cognate tRNA-Arg-ACG that is fully modified with I_34_ but unexpectedly also harbours an uncharacterized methylation at G_18_.^21^ Second, a recent study demonstrated that the formation of m^5^C_38_ by Dnmt2 on Val-AAC depends on the pre-existing A-to-I modification at position 34.^51^ Given that methylation of C_38_ of Asp-GTC and Gly-GCC protect them against endonucleolytic cleavage,^53^ it is possible that hypomodification of Val-AAC at position 34 makes it more vulnerable to such cleavage. Therefore, full characterization of the modification status of each tRNA molecule will be a strong asset to understand the differential downregulation of ADAT2/ADAT3 target tRNAs upon decreased I_34_ levels.

Defects in tRNA modifications lead to either global or codon-specific translational impairment. As the I_34_ resides in the wobble position of the tRNA anticodon, it can have a profound impact upon codon-anticodon recognition and shape the proteome landscape in a codon-specific manner. First, ADAT deficiency in HEK293^27^ cells and *Neurospora*^31^ specifically modulates decoding rates for ADAT-dependent codons, meaning codons that are only read by I_34_ tRNAs (C-ending codons), leading to impaired translation of specific proteins containing regions encoded by sequences enriched in those codons. Interestingly, our computational analysis of synonymous codon usage revealed that genes involved in neuronal migration preferentially use codons that are only read by I_34_ tRNAs (C-ending codons) compared with the whole genome. This preference is statistically significant (*P* < 0.05) in four out of the eight ANN-tRNA families and show a trending significance in the remaining four families (*P* < 0.1) (Supplementary Fig. 6A). Second, computation of tRNA gene usage has suggested that the specific enrichment of tRNA-Ala-AGC anticodon in neurons drives an increased translational efficiency at Ala codons including GCC codons that are read by tRNA-Ala-I_34_GC.^49^ Given that deamination of tRNA-Ala-AGC is the most severely affected in patient cells (Fig. 5A), this raises the possibility that defects in decoding Ala codons contribute to neurodevelopmental phenotypes associated with the mutant ADAT2/ADAT3 complex. Third, as tRNA I_34_ modification has been shown *in vitro* to be a critical determinant for recognition by the yeast isoleucyl-tRNA synthetase,^54^ defects in the I_34_ formation observed upon ADAT3 mutation might lead to accumulation of uncharged or mischarged tRNAs and subsequent translational stalling or errors, directly affecting homeostasis of proteins enriched in a specific amino acid. Additionally, genes involved in neuronal migration, compared with the brain cortex whole genome, showed an enrichment in codons read by tRNAs found to be dysregulated upon *ADAT3* mutation (Supplementary Fig. 6B). This enrichment was observed even when comparing these genes to those associated with generalized cellular migration (Supplementary Fig. 6B). This suggests that a specific translational impairment of neuronal migration genes, which are enriched for ADAT-dependent codons, rather than a global translation defect, likely underlies the defective neuronal migration. Yet, the identity of affected proteins and how mutant ADAT3 impairs the efficiency and accuracy of their translation remain to be identified to better understand the sensitivity of the brain to ADAT3 loss of function.

Altogether, our results demonstrate that maintaining a proper level of ADAT2/ADAT3 complex activity and subsequent level of I_34_ modification is critical for cerebral cortex development. We propose a model in which the variant alters both the solubility and the activity of the complex so that it dictates the severity of the phenotype induced by the given variant. Whether or not the threshold activity of the complexes required for proper migration or for other developmental process is the same remains to be tested. Overall, our results raise the possibility of a threshold of activity below which the tRNAs modification (I_34_) would be compatible with life but not sufficient to ensure protein demand during brain development, leading to neurodevelopmental disorders.

## Supplementary Material

awaf109_Supplementary_Data

## Data Availability

All other relevant data included in the article are available from the authors upon request. High-throughput sequencing data has been deposited in the Gene Expression Omnibus Database (GSE278536, https://www.ncbi.nlm.nih.gov/geo/query/acc.cgi?acc=GSE278536). The mADAT2/ADT3-A180V structure has been deposited in the Protein Data Bank under the PDB ID 9HMM (https://www.rcsb.org/structure/9HMM).
