# Supplementary material for: ADAT3 variants disrupt the activity of the ADAT tRNA deaminase complex and impair neuronal migration
Source: Brain. 2025 Mar 22;148(9):3407–21. doi: 10.1093/brain/awaf109 (PMC12404733; doi:10.1093/brain/awaf109)
Supplement: awaf109_Supplementary_Data [file awaf109_supplementary_data.zip › brain-2024-02790-File009.pdf]

Supplementary information

ADAT3 variants disrupt the activity of the ADAT tRNA deaminase complex and impair neuronal migration.

Del-Pozo-Rodriguez et al.

Content:

- **Supplementary note 1**: Clinical features of newly-identified patients with *ADAT3* variants.
- **Supplementary Figure 1**: Ubiquitous knockdown of *Adat3* and *Wdr4* recapitulates their neuron specific knockdown.
- **Supplementary Figure 2.** Effect of the V144M and A196V/L ADAT3 mutants at the transcript level.
- **Supplementary Figure 3**: Position of the V144M and A106V/L variants in mouse ADAT3 and their effect on enzymatic activity.
- **Supplementary Figure 4**: Deamination and abundance of ADAT2/ADAT3 target tRNAs in patient cells.
- **Supplementary Figure 5**: Overexpression of WT and ADAT3 variants does not impair neuronal migration
- **Supplementary Figure 6.** ADAT-dependent codons are enriched in neuronal migration genes
- **Supplementary material and methods**
- **Supplementary Table 2:** Blast analysis of the miRNAs used in this work.
- **Supplementary Table 4:** Crystallographic table.
- **Supplementary Table 6:** List of sense and antisense oligos used in this work.
- **Supplementary Table 7:** Condition of *in utero* electroporation used in this work.
- **Supplementary Table 8:** List of primary and secondary antibodies used in this work.
- **All full-length unedited Western Blot images**

Supplementary Note 1: Clinical features of newly-identified patients with *ADAT3* variants

Patient 1 (c.430G>A, p.Val144Met)

Patient 1 is a 8 year-old male with uncomplicated term birth and product of consanguinity whose core phenotype included global developmental delay and now intellectual disability with spastic diplegia. He has hypertonicity and hyperreflexia of the lower extremities with axial hypotonia. He has craniofacial dysmorphisms with prominent ears and wide nasal bridge. MR imaging is notable for dysplastic appearance to the corpus callosum with decreased thickness of the posterior callosal body and splenium and blunted rostrum. Treatment for his neurologic comorbidities is largely supportive.

Patient 2 (c.430G>A, p.Val144Met)

Patient 2 is a 4 years and 4 months old female patient, born to a consanguineous parent (2nd degree). She was born at term by normal spontaneous vaginal delivery. At 4 days, she was admitted to the neonatal intensive care unit (NICU) due to premature rupture of membrane. She has one healthy older sibling and one cousin presenting with global developmental delay and microcephaly. She started walking at 3 years. She speaks only few words and started speech therapy just before she turned 4 years-old. All metabolic functions (CPK, amino acids and acylcarnitine profile by tandem mass-spectrometry, urine organic acids profile, liver and kidney function tests) were normal. On examination at 1 year old height and weight were respectively 70cm (-1.28 SD) and 6.5 kg (-3.8 SD). At 4 years and 4 months of age, height was 90cm (-2.93 SD), weight was 10kg (-3.71 SD) and head circumference was 46cm (-1.19 SD), there was hypotonia, hyperreflexia and dystonic movements of both hands. Brain MRI performed at 1year and 5 months showed prominent left temporal surface subarachnoid space and parieto-occipital thickness.

Patients 3 and 4 (c.430G>A, p.Val144Met)

Patient 4 is a 3.5 years old female patient, born to a consanguineous parent. The mother suffered from albuminuria but did not show hypertension. She was born by c-section and presented for neonatal jaundice that lasted for 9 days. Patient 4 presented with global developmental delay and behavioural difficulties (hyperactivity), as well as recurrent episodes of generalized tonic clonic seizures from age 2.5

years. Neuroimaging showed subdural hematomas and the patient was found to have thrombocytopenia. Clinical examination showed hypertonia, hyperreflexia with positive Babinski sign. At age 3.5 years, the patient was found to have short stature (height 3rd percentile) and microcephaly. Weight was at 12th percentile. Her 4.5 years-old affected brother is male presented with global developmental delay, behavioural difficulties (hyperactivity) and abnormal movements. Clinical examination showed microcephaly, hypertonia, hyperreflexia with positive Babinski sign.

Patients 5 and 6 (c.430G>A, p.Val144Met)

Patient 5 is a male, born to non-consanguineous Egyptian parents by Cesarean section following a full-term pregnancy. There was meconium-stained amniotic fluid and he was admitted to the neonatal intensive care unit after birth, though discharged on day 1. The following day he developed cyanosis and was readmitted for 15 days but was never mechanically ventilated. The parents noticed a delay in achievement of early developmental milestones, as he was not rolling or sitting at 5 months. At 8 months, he had generalized tonic seizures, which were controlled with valproic acid. On examination at 3 years 10 months of age, head circumference was 47cm (-2.1 SD), there was plagiocephaly, hypotonia and brisk reflexes, cranial nerves were intact with poor eye contact, and a fundus examination was normal. The parents reported he was not acquiring new words, only used “mama” and “dada”, had frequent constipation, and that he was hyperactive and had stranger anxiety. Normal laboratory investigations included: karyotype, CBC, blood chemistry, ammonia and lactate, CPK, amino acids and acylcarnitine profile by tandem mass-spectrometry, urine organic acids profile, liver and kidney function tests, and urine guanidino acetate/creatine. Brain MRI at 2 years 7 months showed a small brain with appearance of bilateral injury and abnormal gyrification that is worse in the posterior perisylvian regions, left more so than right. There was cortical atrophy in the right parietal lobe and bilateral occipital poles with astrogliosis in subcortical white matter. There were large sylvian fissures, a thin corpus callosum, small hippocampal commissure, small thalami, enlarged cerebral ventricles, diffusely diminished white matter volume and near absent myelination. The orbits, brainstem, pons, and cerebellar vermis and hemispheres appeared unremarkable. An EEG at 2 years 11 months showed left temporal epileptogenic discharge. Since then,

Patient 5 has been treated with sodium valproate treatment. An echocardiogram revealed ASD secundum.

Patient 6, who is the sister of Patient 5, was diagnosed prenatally with the same homozygous variant as her brother. She was delivered via cesarean section without complications. Shortly after birth, she displayed delays in both motor and mental developmental milestones. At one year old, she began experiencing seizures in the form of recurrent tonic fits, which were effectively managed with leviteracetam. Upon clinical examination, she exhibited generalized hypotonia in both her trunk and limbs, had a head circumference of 44.5 cm, and displayed dysmorphic facial features including frontal bossing, synophrys, low-set ears, hypertelorism, a long philtrum, and elongated facial structure. She also had an upward flexion deformity of the upper limbs with both hands clenched into fists. By the age of 1.5 years, she required support to sit and did not engage in babbling or verbal communication. Her brain MRI revealed generalized cortical atrophy more pronounced in the frontotemporal area and mild ventricular ectasia.

Patient 7 (c.430G>A, p.Val144Met)

Patient 7 is a 1 year 9-month-old male, born to a consanguineous parent, with no relevant family history. He presented with global developmental delay and failure to thrive. Clinical examination showed strabismus, hypotonia, intact deep reflexes. Dysmorphic features including prominent forehead, broad forehead, broad nasal bridge, telecanthus, epicanthus, arching eyebrows, downward slanting palpebral fissures, thin upper vermilion border, micrognathia, large ears, wide mouth and teeth abnormalities. At 1y10m, the patient was found to have short stature height 77 cm (-2.5 SD), underweight weight 8.6 kg (-3.6 SD) and microcephaly (head circumference age 17 months 44 cm (-2.7 SD)). Head CT showed bilateral oto-mastoiditis, ethmoid-maxillary sinusitis and enlarged nasopharyngeal adenoid. Brain MRI performed at 2y1m did not show any brain anomalies.

Patient 8 (c.430G>A, p.Val144Met)

Patient 8 is a 1 year old female, born to consanguineous parents with negative family history. She is a product of late preterm 36 weeks born via normal spontaneous vaginal delivery, she was admitted to the

neonatal intensive care unit for around 6 weeks due to recurrent cyanosis and denaturation, she required noninvasive mechanical ventilation. She presented with hypotonia, failure to anf and global developmental delay. Clinical examination showed dysmorphic features in form of prominent forehead, epicanthus, upslanting palpebral fissures, depressed nasal bridge and low- set ears. Neuroimaging which includes brain MRI and MRS were both normal.

Patient 9 (c.430G>A, p.Val144Met)

Patient 9 is a Persian 16 years old female, born to first cousin parents. Preterm delivery occurred through cesarean section because of nuchal cord at 36 weeks of pregnancy. She represented developmental delay since infancy and was able to sit after 18 months and could walk at the age of 3 years. Clinical examination showed hypotonia, hypertelorism, low set ear and hypotonic distal extremities. Hearing and vision were normal and there was no microcephaly.

The patient showed paranasal sinus inflammation, adenoid tissue hypertrophy and cavum septum pellucidum according to brain MRI imaging at 10 years and the EEG performed at 14 years was normal. No chromosomal abnormalities were found in the conventional karyotyping of the patient.

Patients 10 and 11 (c.587C>T ; c586_587delinsTT, p.Ala196Val; p.Ala196Leu)

Patient 10 was born via induction for intrauterine growth restriction at 34w GA and is the product of a pregnancy complicated by maternal preeclampsia and intrauterine growth restriction. Apgar scores were 2 and 8, and she spent a month in the neonatal intensive care unit for feeding difficulties and failure to thrive. She was noted in the first year of life to have developmental delay, hypotonia, intestinal malrotation and diaphragmatic hernia requiring surgical repair, as well as mild facial dysmorphisms including a bulbous nose, wide nasal bridge, thin upper lip, high-arched palate, and epicanthal folds. Her motor milestones were delayed, and she sat unassisted at 12m, crawled at 2y, and walked at 2y11m. Clinical examination confirmed microcephaly at 23 months of age. Prior to that, the HC was measuring 5th centile, but noted to be comparatively large or her body as height and weight were measuring <3rd centile.

The following studies was done with normal results: CSF and blood lactate, CSF neurotransmitters, 7-dehydrocholesterol, acylcarnitine panel, IGF1, biotinidase level, serum amino acids, urine organic acids, carbohydrate deficient transferring levels, VLCFAs, creatine kinase, ammonia, echocardiogram, skeletal survey, renal ultrasound, and brain MRI. The following genetic testing was normal: karyotype (46 XX), chromosomal microarray, ERCC6 and ERCC8 testing, MECP2 sequencing, CDKL5 sequencing. Clinical exome sequencing (ES) identified two variants of unknown significance (NEB (NM_001164508.2; c.7845T>A; p.Q2616L); PCCB (NM_000532.5, c.1229G>A; p.R410Q) neither of which were felt to be clinically relevant. Maternal ancestry is German/Irish, paternal ancestry is German/Eastern European, and consanguinity is denied.

Patient 11 is the younger full brother of patient 10 and presented with a similar clinical course of developmental delays and minor facial dysmorphisms. He born via induction for intrauterine growth gestriction at 35w GA and is the product of a pregnancy complicated by maternal preeclampsia and intrauterine growth gestriction. Apgar scores were 6 and 9, and he spent three weeks in the neonatal intensive care unit for transient hypoglycemia. His motor milestones were delayed and he sat unassisted at 12m, did not crawl, and walked at 3y.

Clinical ES was performed for Patient 11 at a different laboratory following his sister's nondiagnostic ES. His ES identified compound heterozygous variants in ADAT3 (c.586_587delinsTT, paternal; c.587C>T, maternal). Targeted reanalysis of Patient 10's clinical ES confirmed the presence of both ADAT3 variants in her as well. Research genome sequencing (GS) was performed on all four family members and similarly identified the compound heterozygous ADAT3 variants in both children with biparental inheritance.

At most recent clinical exam, Patient 10 was 13y and Patient 11 was 9y. Both have severe intellectual disability. While neither child has expressive language, they can communicate via limited signs, sight words, and an electronic interactive speech system. While motor milestones were delayed, both ambulate independently. Both have similar facial dysmorphisms including high forehead, hypertelorism, telecanthus, and a long, smooth philtrum.

Patient 12 (c.430G>A, p.Val144Met)

Patient 12 is an 11-year-old girl born to first degree consanguineous cousins with positive family history of similarly affected brother with similar presentation of global developmental delay and intellectual disability. She was referred to the hospital with history of 2 times sudden syncopal attack in the form of sudden collapse, loss of consciousness, uprolling of the eyes lasting for 2 minutes with postictal tiredness and vomiting. One attack was at the age of 10 years old and the other was 10 months after. She is a product of normal spontaneous vaginal delivery, full term, uneventful pregnancy, discharged home in good condition. She has history of surgical correction of the hip at the age of 8 years. The family noted delays in her motor and social developmental skills: she walked at the age of 6 years old, she could not climb the stairs and could not ride a bicycle. There were also notable delays in her language and communication. Her parameters at the age of 10.5 years were (Weight 25th centile, height 25th centile, and head circumference 10th centile). She has mild divergent squint of right eye with head nodding. She also has mild dysmorphism (frontal bossing and elongated face). She has abnormal gait. She had normal tone with increased reflexes. She had normal ECG, normal echocardiography, and unremarkable brain MRI.

Patient 13 (c.430G>A, p.Val144Met)

This 13-month-old baby boy was referred to our hospital for surgery for the correction of tetralogy of Fallot and elevated CK. The child was born as a result of in vitro fertilization delivery and is molecularly diagnosed with duchenne muscular dystrophy (a hemizygous deletion affecting exon 12 and exon 13 of DMD) and Neurodevelopmental disorder with brain abnormalities, poor growth, and dysmorphic facies (a homozygous variant in ADAT3). He has shown some progress in his motor development whereby he is rolling over, reaching out but has not been able to sit yet at the age of 13 months and still cannot walk or crawl. Parents report that he is social, but does not have any clear words (only vocalizes). There is mild arthrogryposis at elbows and knees. He also has some dysmorphic features with frontal bossing, hypertelorism, depressed nasal bridge, and deep-seated eye sockets). The patient has mild hypertonic in both lower limbs. Normal tone in both upper limbs with normal bowel reflexes. His brain MRI was unremarkable.

Patient 14 (c.430G>A, p.Val144Met)

Patient 14 is a 6-year-old female who was referred for evaluation of developmental delay, failure to thrive and microcephaly. She was born post-term with pregnancy induced hypertension and spent 4 days in NICU on phototherapy. Subsequently, her milestones were slow in all areas and she was failure to thrive. She has speech delays; however, she follows commands and she has receptive language function. Brain MRI revealed no structural abnormality. Her ultrasound of the abdomen was normal and echocardiogram was normal. She continues to grow less than the 5th percentile for both height and weight. She can feed herself with the spoon and drinks from a cup. She can help in dressing herself but is not toileting. She runs and walks but is somewhat fragile and her fine motor control is not to her age. She is social with good eye contact and enjoys interacting with others at home.

Patient 15 (c.430G>A, p.Val144Met)

Patient 15 is a 9 years old boy who is a product of a full term normal spontaneous vertex delivery with no NICU admission. His birth weight was 2.5 kg. He was referred for a dislocated left hip and clubfoot, left foot. Parents are consanguineous and he has two healthy siblings but there is a strong family history of development dysplasia of the hip from the paternal side. He was also found to be delayed globally; he is alert but not very responsive, he makes constant sounds which are repetitive but has no real words. He has awkward gait which could be secondary to his hip dysplasia. He feeds himself with finger food and drinks from a cup. He is not toileting or dressing and he follows some few simple commands. He has severe cognitive delay, severe linguistic and social impairments, and better motor skills. The patient looked dysmorphic but has no history of seizure or any abnormal movement. MRI brain was negative.

Patient 16 (c.430G>A, p.Val144Met)

Patient 16 is a 2 years old girl with history of NICU admission for 2 months due to respiratory distress, urosepsis, hypokalemia and proved seizure. She has mild global developmental delay; she sits without support, stand with support, she can hold and transfer objects, she has no clear words. Current medication: Vigabatrin, - Valproic acid, and carnitine. Seizure onset was at the age of 10 months and she had abnormal EEG study due to slow background activity with intermittent slowing along with spike and

spike and wave generalized which is suggestive of epileptic encephalopathy. Her brain MRI was remarkable for diffuse decreased parenchymal volume with CSF spaces widening, predominantly in left cerebral hemisphere and hypoplastic corpus callosum. Her growth parameters are (Height 80cm, weight 8.9kg, HC 44cm). She is dysmorphic she can move all limbs freely. Tone was difficult to assess along with reflexes due to constant moments.

Patient 17 (c.430G>A, p.Val144Met)

Patient 17 is an 8-year-old girl with microcephaly, developmental delay and speech delay. She is a full term product of normal spontaneous vaginal delivery with birthweight of 2.5 kg with no NICU admission. The mother had a history of bleeding in the 1st trimester. The parents are 1st degree cousins. She has other 2 sisters and 2 brothers; and one of her sisters had a history of thyroid enlargement. There was a 1 history of abortion at 2 months. She can stand and crawl on furniture. She has a firmer grasp, and poor eye contact. There was no history of seizure and she was not on any medications. Audiology was normal. VEPs ophthalmological examination was normal but she has hypothyroidism. Her EEG was unremarkable and her MRI brain showed no structural cerebral abnormalities. Her growth parameters were below the 3rd centile. She is dysmorphic (prominent forehead, upturned nose, smooth philtrum) and she has axial hypotonia, pectus excavetum, and lower limb spasticity.

Patient 18 (c.430G>A, p.Val144Met)

Patient 18 is a 6-year-old boy with dysmorphism and developmental delay. He is a product of consanguineous marriage. He has a younger brother who is healthy. No abortions or neonatal death. No family history of similar illness, developmental delay, or epilepsy. He was noted to be floppy since discharge from nursery. He also showed delay in gaining millstone compared to children in his age. Family sought medical advice at age of 8 months when they noted that he has no head control. They also reported staring spells around once a month during which his attention can be regained. He is a product of an uneventful pregnancy with good fetal movement and normal antenatal ultrasound. He was delivered at full term via normal spontaneous vaginal delivery without complications. He cried immediately. His birth weight was 2.3 kg. He controlled his head at 12 months of age, sat without support at 18 months, and

walked independently at 3 years. He can go up and down the stairs with assistance. He started cooing at 8 months first word was said at 12 months. He has interactive play. He is toilet trained, needs minimal assistance with dressing and eating. He had one undescended testis. Head circumference was 49.6 cm on the 5th percentile. He had normal head shape. Height and weight were below the 3rd percentile. Skin showed no neurocutaneous stigmata. He had normal axial tone with decrease appendicular tone with tight Achilles tendon bilaterally. Power was 5/5 in upper and lower limbs. Deep tendon reflexes were +2 all over and symmetric with down going plantar response bilaterally. Coordination was normal. Gait was imbalance due to foot deformity. Musculoskeletal system showed bilateral talipes equinovarus. His brain MRI was unremarkable, however, echocardiography showed ASD secundum. EEG showed recurrent slow wave at frontocentral region.

Patient 19 (c.430G>A, p.Val144Met)

Patient 19 is an 8 years 2 months old boy with microcephaly, global developmental delay, speech delay, and seizures. His ophthalmological examination revealed severe esotropia, high myopia (-7.5 and -8.5), and astigmatism; He does not run and only babbles. His growth parameters are delayed (Height: 102.5cm (-4.8SD), weight 12.7kg (-7.7SD), head circumference 49.5cm (5-10th)). He has dysmorphic features (smooth philtrum, severe esotropia). He is very apprehensive and difficult to examine. His brain MRI revealed abnormal shape of splenium, inferior vermis hypoplasia, and abnormality of the corpus callosum.

Patient 20 (c.430G>A, p.Val144Met)

Patient 20 is a 12-year-old boy with global developmental delay, severe intellectual disability, bilateral undescended testes, proximal hypospadias, and he is thought to be on the autism spectrum. He underwent orchidopexy at the age of 6y and underwent first-stage hypospadias repair at the age of 10y. He had developmental delays; walked and sat at the age of 7y and at the age of 12y, he only babbles and does not engage in verbal communication.

Patient 21 (c.430G>A, p.Val144Met)

Patient 21 is a 3-year-old boy with global developmental delay, severe intellectual disability, microcephaly, bilateral abnormal foot morphology with vertical talus and short foot and lower and upper limb spasticity. He had developmental delays and failure to thrive; sat at the age of 14m and is unable to walk, he only babbles and does not engage in verbal communication. MRI at age of 11m revealed microcephaly and generalized reduction in white matter volume, generalized thinning of fully formed corpus callosum and delayed myelination. The EEG performed at 11 months was normal. Bilateral Inguinal hernia, hypoplasia and dysmorphic facial features but no sign of microcephaly were observed at birth.

Supplementary Figure 1. Ubiquitous knockdown of *Adat3* recapitulates the neuron specific knockdown.

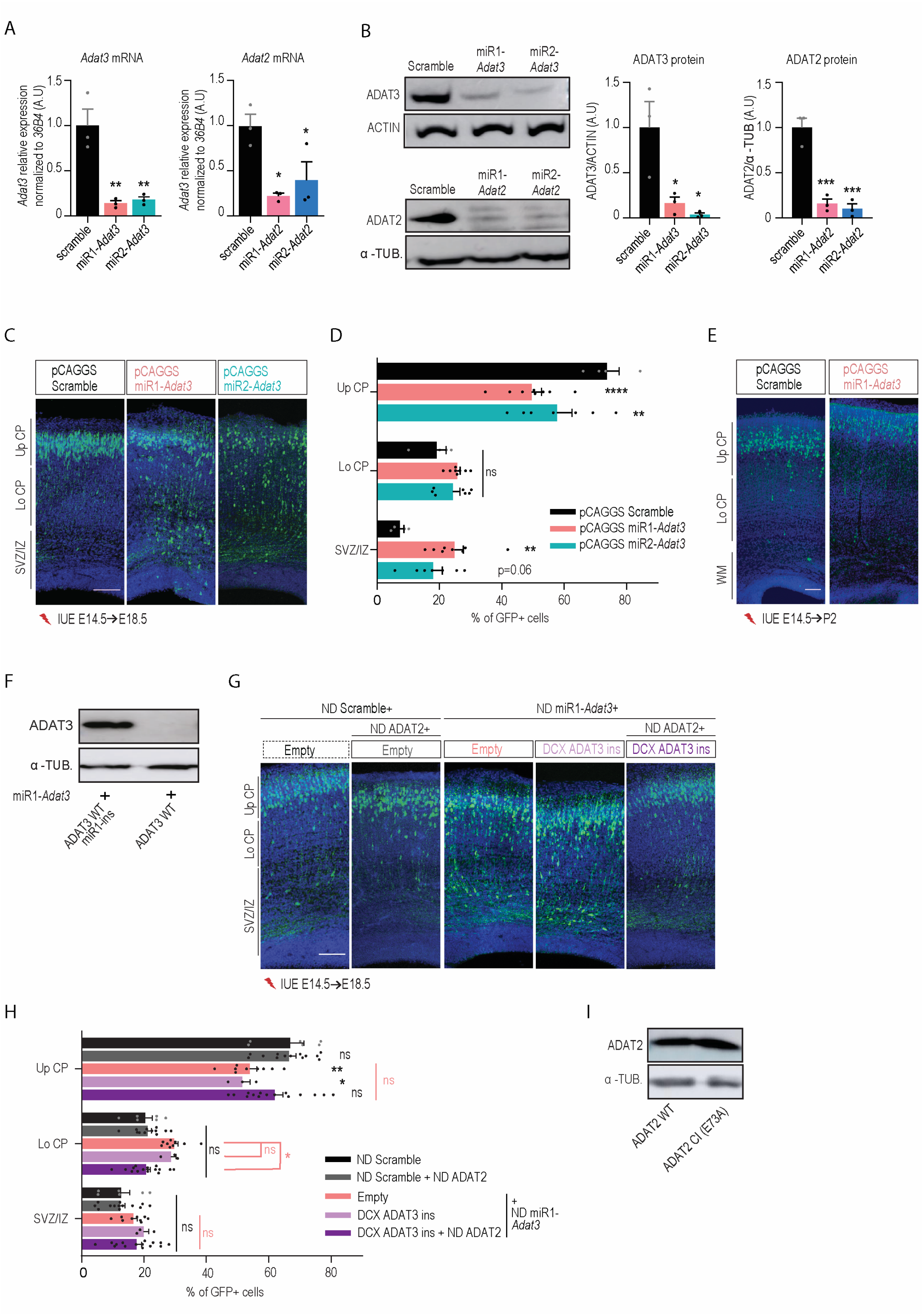

(**A-B**) Knockdown efficiency of *Adat3* and *Adat2* miRNAs assessed by (**A**) RT-qPCR and (**B**) Western blot analysis performed in HEK cells after transfection of pCAGGS-driven miRNAs. Data (n=3 for each condition) are represented as means ± S.E.M. Significance was calculated by one-way ANOVA (Bonferroni's multiple comparisons test), *P < 0.05; **P < 0.005; ***P<0.0005. α-TUBULIN (α-TUB) or ACTIN are used as a protein leading control. (**C**) Coronal sections of E18.5 mouse cortices electroporated at E14.5 with pCAGGS scramble or two distinct pCAGGS-*Adat3* miRNAs (miR1 and miR2) together with ND-GFP. GFP-positive electroporated cells are depicted in green. Nuclei are stained with DAPI. Scale bar, 100 µm. (**D**) Percentage (means ± S.E.M.) of the positive electroporated cells (GFP+, green) in upper (Up CP) and lower (Lo CP) cortical plate, intermediate (IZ) and subventricular zone (SVZ) showing the faulty migration upon ubiquitous silencing of *Adat3*. Data were analyzed by two-way ANOVA (Bonferroni's multiple comparisons test). Number of embryos analyzed: pCAGGS Scramble, n=4; pCAGGs miR1-*Adat3*, n=8; pCAGGS miR2-*Adat3*, n=7. ns non-significant; **P < 0.005; ****P < 0.0001. (**E**) Coronal sections of P2 mouse cortices electroporated at E14.5 with scramble or miR1-*Adat3* under the pCAGGS ubiquitous promoter together with ND-GFP. GFP-positive electroporated cells are depicted in green. Nuclei are stained with DAPI. *Adat3* deficient neurons reach their final position by P2 indicating a transient rather a permanent arrest of migration upon loss of *Adat3.* Scale bar, 100 µm. (**F, I**) Western blot analysis performed in N2A cells to confirm (**F**) the insensitivity of ADAT3 WT miR1 insensitive (ADAT3 WT miR1-ins) construct against knockdown by miR1-*Adat3* and (**I**) the correct expression of ADAT2 catalytic inactive (CI) constructs. α-TUBULIN (α-TUB) is used as a protein loading control. (**G**) Coronal sections of E18.5 mouse cortices electroporated at E14.5 with the indicated constructs. GFP-positive electroporated cells are depicted in green. Nuclei are stained with DAPI. Scale bar, 100 µm. (**H**) Percentage (means ± S.E.M.) of electroporated cells in upper (Up CP) and lower (Lo CP) cortical plate, intermediate (IZ) and subventricular zone (SVZ) showing that expression of ND-ADAT2 together with scramble does not lead to any phenotype and that migration defects upon loss of *Adat3* are rescued only when ND-ADAT2 is co-expressed together with DCX-ADAT3. Data were analyzed by two-way ANOVA (Bonferroni's multiple comparisons test). Number of embryos analyzed: ND Scramble, n =6; ND Scramble + ND ADAT2, n=13; ND miR1-*Adat3* + Empty, n=9; ND miR1-*Adat3* + DCX ADAT3, n=3; ND miR1-*Adat3* + DCX ADAT3 + ND ADAT2, n=15. ns non-significant; *P < 0.05; **P < 0.005. ins: insensitive.

Supplementary Figure 2. Effect of the V144M and A196V/L ADAT3 mutants at the transcript level.

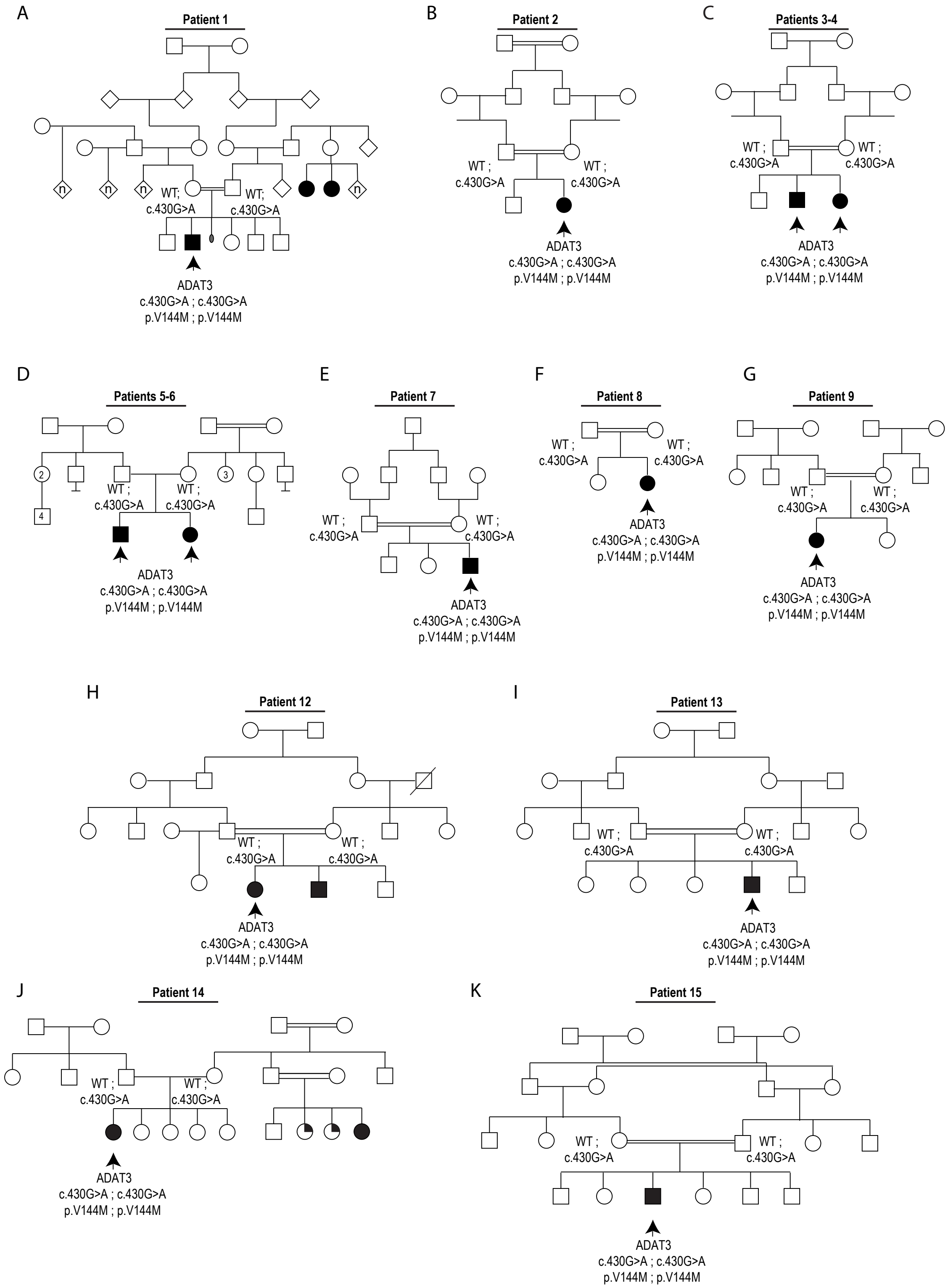

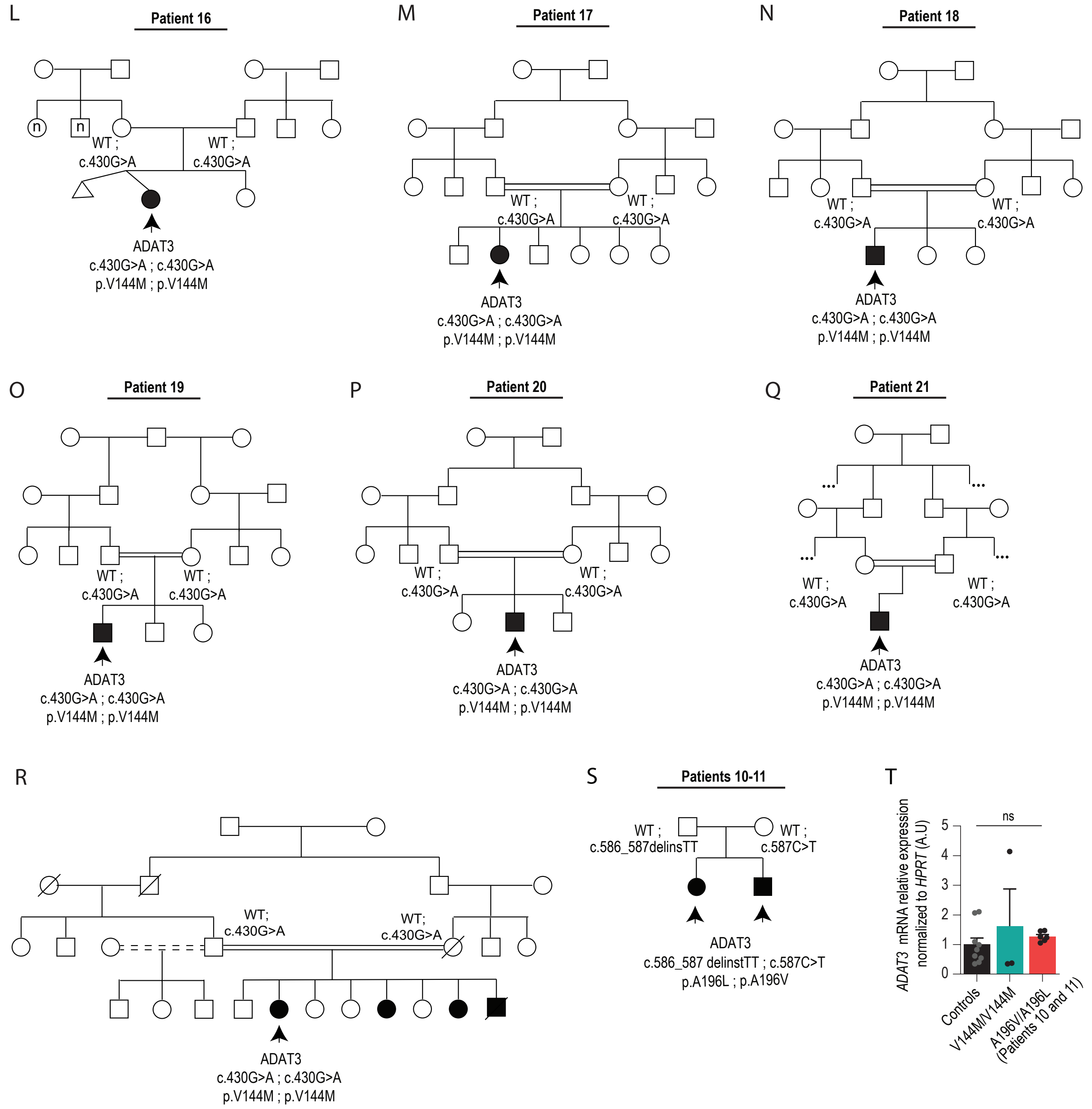

(A-Q) Pedigrees of newly identified patients with *ADAT3* variants. (**R-S**) Pedigrees of previously published patients with the p.V144M/p.V144M[1] and p.A196V/p.A196L ADAT3 (Patients 10 and 11)[2] variants from whom the LCLs were derived to analyze the effect of p.V144M/p.V144M and p.A196V/p.A196L variants. (**T**) RT-qPCR analyses showing unchanged *ADAT3* mRNA levels in patients with p.V144M/p.V144M and p.A196V/p.A196L variants (Patients 10 and 11) in comparison to controls (Controls, n=9; V144M/V144M, n=3 and A196V/A196L, n=6 (3 of each Patient)). *HPRT* is used for normalization. Data were analyzed by one-way ANOVA (Bonferroni's multiple comparisons test). ns non-significant.

Supplementary Figure 3. Position of the V144M and A106V/L variants in mouse ADAT3 and their effect on enzymatic activity.

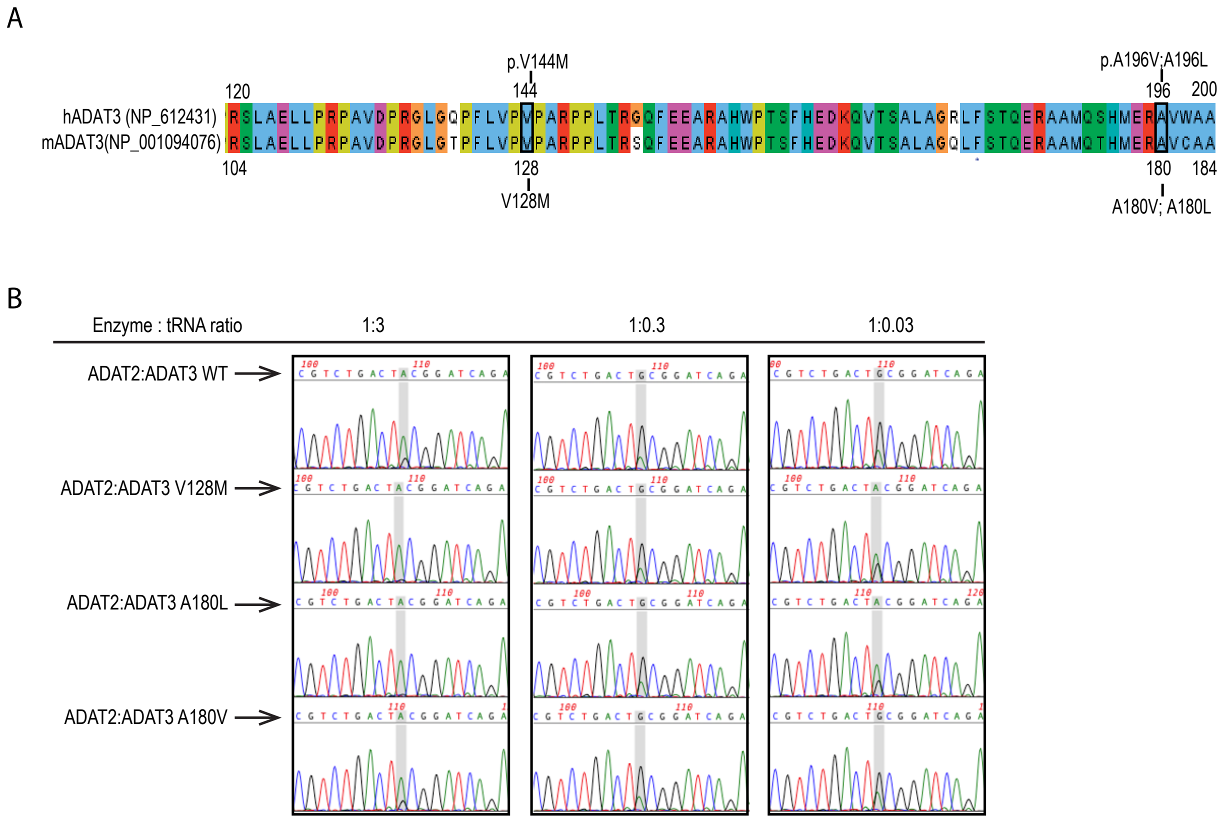

(**A**) Alignment of ADAT3 protein across human and mouse showing the conservation of the p.V144 (corresponding to p.V128 in mouse) and p.A196 (corresponding to p.A180 in mouse) residues. (**B**) Sequencing chromatogram analysis of RT-PCR products amplified from *in vitro* transcribed tRNA-Arg-ACG after incubation with different amounts of purified recombinant WT or mutant ADAT2/ADAT3 complexes. The wobble adenosine/inosine position is highlighted in gray. Inosine is read as G.

Supplementary Figure 4. Deamination and abundance of ADAT2/ADAT3 target tRNAs in patient cells.

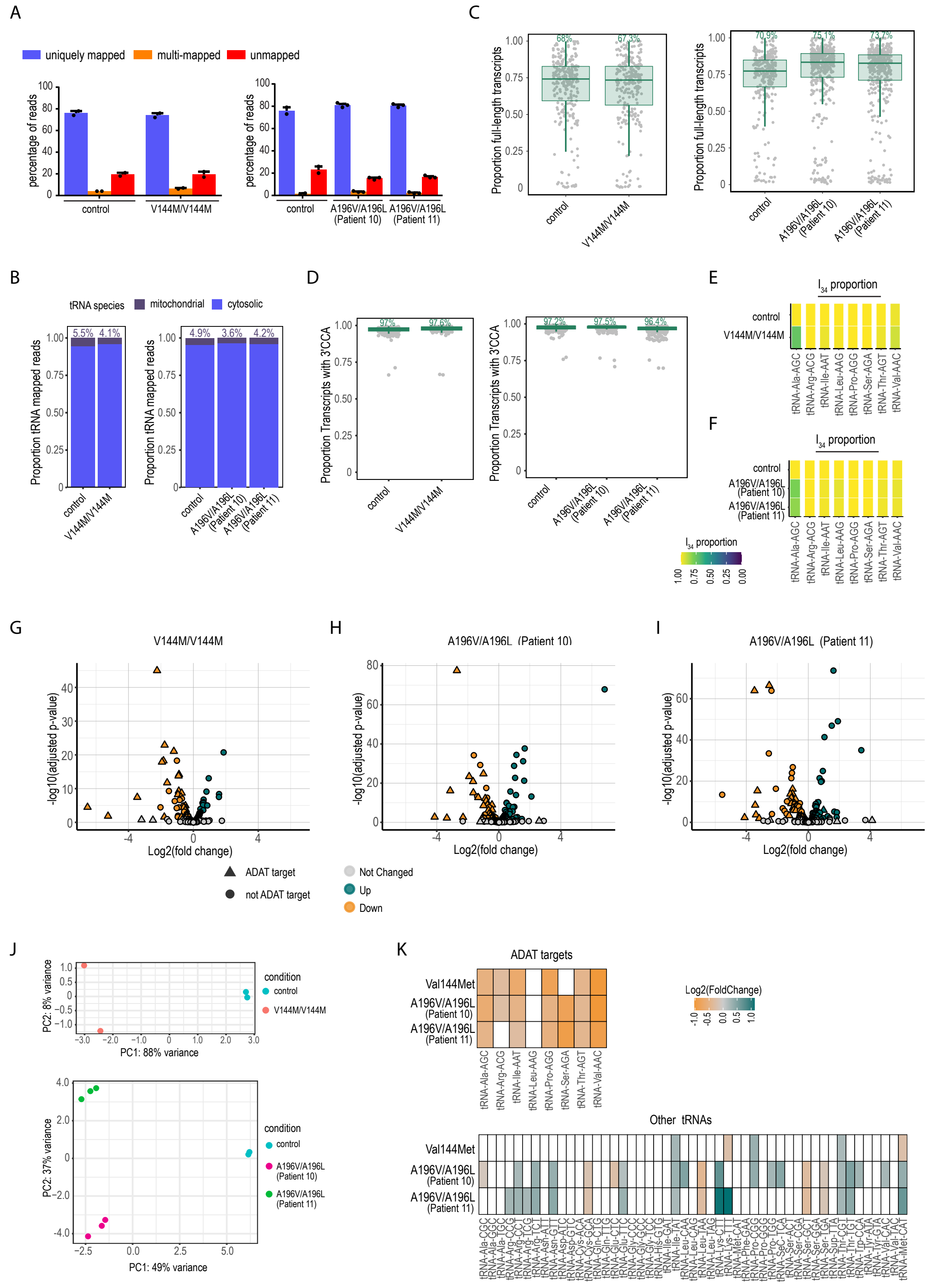

(**A**) Alignment statistics for mim-tRNAseq reads. Bars are mean values per cell line. (Controls, n=2; V144M/V144M, n=2; A196V/A196L (Patient 10) n=3; A196V/A196L (Patient 11) n=3). (**B**) Bar plot of cytosolic and mitochondrial tRNA read fractions per cell line. Bars: mean (Controls, n=2; V144M/V144M, n=2; A196V/A196L (Patient 10) n=3; A196V/A196L (Patient 11) n=3), percentages: mean mitochondrial fraction. (**C-D**) Box plots of (**C**) full-length read fraction and (**D**) full 3′-CCA end fraction per tRNA transcript (Controls, n=2; V144M/V144M, n=2; A196V/A196L (Patient 10) n=3; A196V/A196L (Patient 11) n=3); center line and label: median; box limits: upper and lower quartiles; whiskers: 1.5×interquartile range). **(E-F)** Heatmap showing I_{34} proportion at the anticodon level in ADAT2/ADAT3 target tRNAs in LCLs derived from (**E**) p.V144M/p.V144M and (**F**) p.A196V/p.A196L patients compared to control. (Controls, n=2; V144M/V144M, n=2; A196V/A196L (Patient 10) n=3; A196V/A196L (Patient 11) n=3). (**G-I**) Volcano plot showing the negative $\log_{10}$ adjusted *P*value (p-adj) of all tRNA isodecoders against their $\log_2$ fold change ($\log_2$FC) in LCLs derived from (**G**) p.V144M/p.V144M (n=2) and (**H,I**) p.A196V/p.A196L (Patient 10 (**H**), n=3; Patient 11 (**I**), n=3) compared to control (Controls, n=2). Triangle and circle show ADAT targets and non-target tRNAs respectively. Green, orange and grey represent upregulated, downregulated and unchanged tRNAs respectively based on DESeq2 padj<0.05. (**J**) Principal component analysis (PCA) of count data for tRNA transcripts from DESeq2 for each cell line. PCi axis represents the principal component i and the number indicates the percentage of explained variance associated with this axis. (Controls, n=2; V144M/V144M, n=2; A196V/A196L (Patient 10) n=3; A196V/A196L (Patient 11) n=3) (**K**) Heatmap showing log2 DESeq2 fold change summed by anticodon. White boxes show non-significant ones.

Supplementary Figure 5. Overexpression of WT and ADAT3 variants does not impair neuronal migration.

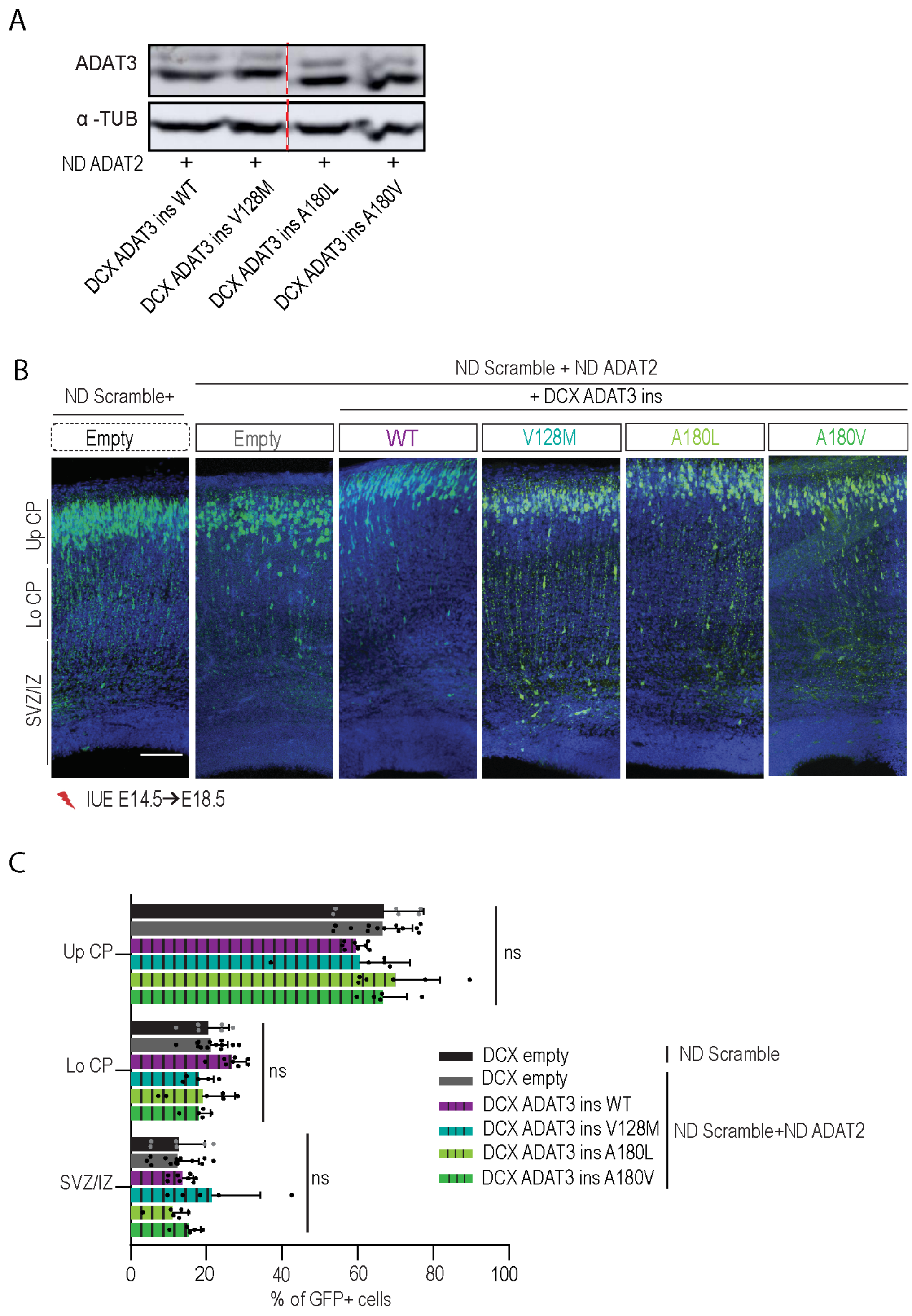

(**A**) Western blot analysis of showing similar expression of ADAT3 WT, V128M, A180L and A180V constructs. Red dashed line shows the position of membrane cut to remove constructs that were not used in this study. α-TUBULIN (α-TUB) is used as a protein loading control. (**B**) Coronal sections of E18.5 mouse cortices electroporated at E14.5 with NeuroD (ND) scramble either alone or together with ND ADAT2 in combination with DCX Empty or DCX ADAT3 WT or DCX ADAT3 miR-1 insensitive (ins) variants. GFP-positive electroporated cells are depicted in green. Nuclei are stained with DAPI. Scale bar, 100 μm. **(C)** Analysis of percentage (means ± S.E.M.) of electroporated cells in upper (Up CP) and lower (Lo CP) cortical plate, intermediate (IZ) and subventricular zone (SVZ) showing no effect on migration upon overexpression of any of the constructs. Data were analyzed by two-way ANOVA (Tukey's multiple comparison test). Number of embryos analyzed: ND Scramble, n =6; ND Scramble + ND ADAT2, n=13; ND Scramble + ND ADAT2+DCX ADAT3 WT, n= 8; ND Scramble + ND ADAT2+DCX ADAT3 V128M, n= 5; ND Scramble + ND ADAT2+DCX ADAT3 A180L, n= 6; ND Scramble + ND ADAT2 + DCX ADAT3 A180V, n=5. ns non-significant.

Supplementary Figure 6. ADAT-dependent codons are enriched in neuronal migration genes

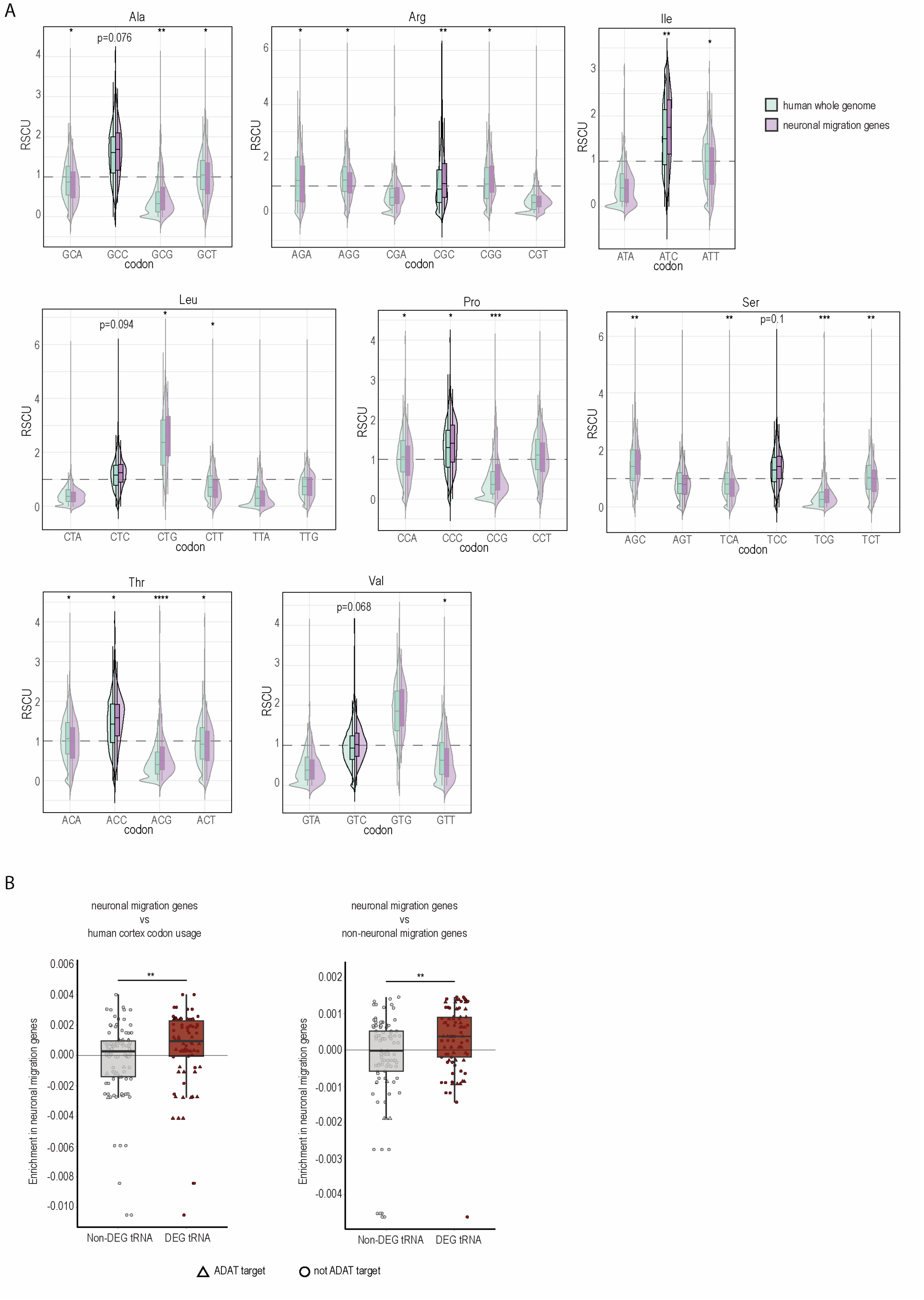

(**A**) Violin and box plots showing relative synonymous codon usage (RSCU) distribution across the whole human genome (light blue) or gene associated to neuronal migration associated (fuchsia) for the ADAT-sensitive codon families. Black stroke indicated codon whose translation is influenced by the presence of Inosine in position 34. Across all 8 codons, half of them show significant preference (Ile-ATC p = 0.003, Arg-CGC p = 0.004, Pro-CCC p = 0.031, Thr-ACC p = 0.038) and the other half show a consistent trend for preference (Val-GTC p = 0.068, Ala-GCC p = 0.076, Leu-CTC p = 0.094, Ser-TCC p = 0.1) according to pair-wise Dunn's test. **P < 0.05; **P < 0.005; ***P < 0.001; ****P < 0.0001. Center line: median; box limits: upper and lower quartiles; whiskers: 1.5×interquartile range). (**B**) Boxplot showing the degree of codon enrichment in human genes associated to neuronal migration in comparison to human genes associated to (right) non-neuronal migration or (left) codon frequency usage of the human brain cortex. Codons are split based on the differential status of their matching anticodon. Codon translated by tRNAs found dysregulated in the mimtRNAseq (red) are significantly more enriched than the ones translated by tRNAs found not changed (gray). Wilcoxon rank sum test, **P < 0.005. Center line: median; box limits: upper and lower quartiles; whiskers: 1.5×interquartile range.

Supplementary Materials and methods

WES and patients

Patient 1

Patient 1 and the parents were part of the IRB (P00032816)-approved Boston Children's Hospital (BCH) CP Sequencing Study and underwent trio-exome sequencing. Full methods regarding DNA isolation, sequencing and variant identification are described in the original paper.[3]

Patient 2

Genomic DNA was extracted from the peripheral blood of Patient 2 and WES was carried in Centogene labs using CentoXome® Solo. DNA was enzymatically fragmented, and target regions (including approximately 41 Mb of the human coding exome (targeting > 98% of the coding RefSeq from the human genome build GRCh37/hg19), as well as the mitochondrial genome were enriched using DNA capture probes. The generated library was sequenced on an Illumina platform to obtain at least 20x coverage depth for > 98% of the targeted bases. An in-house bioinformatics pipeline, including read alignment to GRCh37/hg19 genome assembly and revised Cambridge Reference Sequence (rCRS) of the Human Mitochondrial DNA (NC_012920), variant calling, annotation, and comprehensive variant filtering was applied. All variants with minor allele frequency (MAF) of less than 1% in gnomAD database, and disease-causing variants reported in HGMD®, in ClinVar or in CentoMD® were evaluated. All potential patterns for mode of inheritance were considered and provided family history and clinical information were used to evaluate identified variants with respect to their pathogenicity and disease causality. This study was conducted in accordance with the declaration of Helsinki 1975 for studies involving human participants and was approved by the institutional review board at faculty of medicine, Cairo University (IRB N-486-2023).

Patients 3 and 4

DNA from the probands was subjected to Agilent Sure-Select Human All Exon v2.0 (44Mb target) and Illumina Rapid Capture Enrichment (37Mb target) library preparation and sequenced on Illumina HiSeq 2000 or 4000 instruments. WES was performed on blood-derived DNA from two siblings. This study was approved by the Institutional Review Board at the University of California San Diego Human Research

Protection Program (HRPP), San Diego, USA under protocol #140028 entitled “The Genetics of Childhood Neurological Diseases” with Dr. Gleeson as PI. Variant identification using genome analysis toolkit (GATK) workflow identified variants that were intersected with identity-by-descent blocks from Homozygosity Mapper. Variants were filtered for minor allele frequency (MAF) > 1:1000, PolyPhen-2 scores of < 0.9, or GERP score < 4.5, and runs of homozygosity were defined with Homozygosity Mapper. Potentially deleterious variants were prioritized against an in-house exome database consisting of 10,000 ethnically matched individuals, in addition to publicly available exome datasets, cumulatively numbering over 20,000 individuals. The identified mutation was confirmed by Sanger sequencing and segregation in both parents of the affected children. We prioritized predicted protein frame shift, stop codon, splice defects, and conserved nonsynonymous amino acid substitution mutations [Genomic Evolutionary Rate Profile (GERP) score > 4 or phastCons (genome conservation) score > 0.9]. We excluded variants with an allele frequency of greater than 0.2% in our internal exome database of over 2000 individuals.

Patients 5 and 6

Patient 5 and his family were identified and evaluated in a clinical setting, and biological samples were collected for research purposes after obtaining written informed consent according to protocols approved by Cairo University Faculty of Medicine Research Ethics Committee (N-78-2016) and Boston Children's Hospital Institutional Review Board (05-05-076R). Trio exome sequencing and data processing for the family were done at the Genomics Platform at the Broad Institute of MIT and Harvard. An exome sequencing library was generated using the Illumina Nextera exome capture kit and sequenced using 150 bp paired-end reads to cover >80% of targets at 20X coverage and a mean target coverage of >100X. Exome sequencing data were processed using the standard pipeline with Picard. BWA[4] was used to align reads to hg38, and SNV and insertions/deletions were called using GATK HaplotypeCaller[5] using default filters. Variants were annotated using Variant Effect Predictor and uploaded to seqr[6] for review. While pregnant with Patient 6 (the sister of Patient 5), the mother had prenatal diagnosis by targeted sequencing of the c.430G>A variant performed at Centogene that confirmed the homozygous variant.

Patient 7

Peripheral blood of Patient 7 was collected for DNA extraction after obtaining written informed consent according to protocols approved by Cairo University Faculty of Medicine Research Ethics Committee (N-401-2024). The coding and flanking intronic regions were enriched using in solution hybridization technology and were sequenced using the Illumina HiSeq/NovaSeq system.

Copy number variations (CNV) were computed on uniquely mapping, non-duplicate, high-quality reads using an internally developed method based on sequencing coverage depth. Briefly, we used reference samples to create a model of the expected coverage that represents wet-lab biases as well as inter-sample variation. CNV calling was performed by computing the sample's normalized coverage profile and its deviation from the expected coverage. Genomic regions are called as variants if they deviate significantly from the expected coverage.

Bioinformatics and quality control: The bioinformatics analysis began with quality control of raw sequence reads. Clean sequence reads of each sample were mapped to the human reference genome (GRCh37/hg19). Burrows-Wheeler Aligner (BWA-MEM) software was used for read alignment. Duplicate read marking, local realignment around indels, base quality score recalibration and variant calling were performed using Freebayes. Variant data was annotated with public variant databases (VefAnno, VEP). The sequencing depth and coverage for the tested sample was calculated based on the alignments. The sequencing run included in-process reference sample(s) for quality control, which passed our thresholds for sensitivity and specificity. The patient's sample was subjected to thorough quality control measures as well, after which raw sequence reads were transformed into variants by a proprietary bioinformatics pipeline. Copy number variations (CNVS), defined as single exon or larger deletions or duplications (Del/Dups), were detected from the sequence analysis data using a proprietary bioinformatics pipeline, which processes aligned sequence reads. The difference between observed and expected sequencing depth at the targeted genomic regions was calculated and regions were divided into segments with variable DNA copy number. The expected sequencing depth was obtained by using other samples processed in the same sequence analysis as a guiding reference. Our variant classification follows the INHERITANCE Variant Classification Schemes modified from the ACMG guideline 2015. Minor modifications were made

to increase reproducibility of the variant classification and improve the clinical validity of the report. Likely benign and benign variants were not reported. The pathogenicity potential of the identified variants were assessed by considering the predicted consequence, the biochemical properties of the codon change, the degree of evolutionary conservation as well as the number of reference population databases and mutation databases such as, but not limited to, the gnomAD, ClinVar, HGMD Professional and Alamut Visual. For missense variants, in silico variant prediction tools such as SIFT, PolyPhen, MutationTaster were used to assist with variant classification. In addition, the clinical relevance of any identified CNVs was evaluated by reviewing the relevant literature and databases such as Database of Genomic Variants, EXAC, gnomAD and DECIPHER. The clinical evaluation team assessed the pathogenicity of the identified variants by evaluating the information in the patient referral, reviewing the relevant literature and manually inspecting the sequencing data if needed.

Patient 8

Whole genome sequencing was performed after obtaining written informed consent to perform clinal and genomic studies for research purpose. Genomic DNA was enzymatically fragmented and tagged with Illumina compatible adapter sequences. The libraries were paired-end sequenced on an Illumina platform to yield an average coverage depth of ~ 30x. A bioinformatics pipeline based on the DRAGEN pipeline from Illumina, as well as CENTOGENE's in-house pipeline was applied. The sequencing reads were aligned to the Genome Reference Consortium Human Build 37 (GRCh37/hg19), as well as the revised Cambridge Reference Sequence (rCRS) of the Human Mitochondrial DNA (NC_012920). Sequence variants (SNVs/indels) and copy number variations (CNVs) are called using DRAGEN, Manta and in-house algorithms. Variants with a minor allele frequency (MAF) of less than 1% in gnomAD database, or disease-causing variants reported in HGMD®, in ClinVar or in CENTOGENE's in-house Biodatabank were evaluated. Although the evaluation is focused on coding exons and flanking intronic regions, the complete gene is interrogated for candidate variants with plausible association to the phenotype. All potential modes of inheritance are considered. In addition, the provided clinical information and family history are used to evaluate identified variants with respect to their pathogenicity and disease causality. Variants were categorized into five classes (pathogenic, likely pathogenic, VUS, likely benign, and benign) according to ACMG guidelines for classification of variants in addition to ClinGen recommendations. For detection of

SNVs and indels in the regions targeted for downstream analysis a sensitivity of 99.9%, a specificity of 99.9%, and an accuracy of 99.9% is achieved. CNV detection software has a sensitivity of more than 95%. CENTOGENE has established stringent quality criteria and validation processes for variants detected by NGS. Variants with low sequencing quality and/or unclear zygosity were confirmed by orthogonal methods. Consequently, a specificity of > 99.9% for all reported variants is warranted. Screening of repeat expansions is performed by the Expansion Hunter algorithm for the following genes: AR, ATN1, ATXN1, ATXN2, ATXN3, ATXN7, ATXN8OS, ATXN10, CACNA1A, CNBP, CSTB, C9ORF72, DMPK, FMR1, FXN, HTT, JPH3, NOP56, PABPN1, PHOX2B, PPP2R2B, PRNP and TBP. Screening of uniparental disomy (UPD) is performed using an in-house algorithm for Mendelian inheritance errors (MIE) to detect runs of homozygosity (ROH) for the well-known clinically relevant chromosomal regions (6q24, 7, 11p15.5, 14q32, 15q11q13, 20q13 and 20).

Patient 9

DNA extraction from peripheral blood of Patient 9 was performed using a commercial extraction kit (ROCHE, Germany) according to manufacturer instructions. Exome sequencing was performed using the Agilent SureSelect Target Enrichment V6 Kit, and the resulting library was sequenced on the Illumina HiSeq 2000/2500 platform. Reads were aligned to the hg19 human reference genome assembly and subjected to quality control with BWA and Sam tools. Variant calling for single nucleotide polymorphisms (SNPs) and insertions/deletions (indels) carried out using VarScan v2.3.9. Functional annotation of detected variants was conducted with Wannovar. Variants were filtered focusing on known/most relevant genes based on homozygosity, allelic frequencies in population databases, gene function impact, and predictions from various databases. Pathogenicity of detected variants was evaluated according to the American College of Medical Genetics (ACMG) guidelines. Copy number variations and mitochondrial DNA variants were not examined. Prior to blood sampling, informed consent was obtained from patient's legal guardians according to the protocol approved by Ethics Committee of Mashhad University of Medical Sciences, Mashhad, Iran.

Patients 10 and 11

Peripheral blood of Patients 10 and 11 were collected for establishment of lymphoblastoid cell lines. The study protocol was approved by the Massachusetts General Brigham Institutional Review Board (Protocol

#: 2016P001422) and informed consent was obtained from the participating family. Full methods regarding DNA isolation, sequencing and variant identification are described in the original paper.[2]

Patients 12 to 20

Informed consent was obtained from patient's legal guardians according to the protocol approved by KFSHRC Ethics Committee (REC#2070023). DNA extraction from peripheral blood was performed using a commercial extraction kit (Qiagen, USA) according to manufacturer instructions. Exome sequencing was performed on genomic DNA using the Agilent SureSelect Target Enrichment workflow to capture regions of interest from a DNA fragment library. The whole exome is sequenced on the Illumina HiSeq 2500 with a minimum coverage of 30X. Reads were aligned to the human genome build UCSC hg19 genome assembly and an in-house pipeline was used to compare the proband's sequence to the reference sequence. Coverage and quality for targeted coding exons were assessed. Analysis of Exome data was performed as described previously.[7] Identified variants were classified according to the American College of Medical Genetics (ACMG) guidelines.

Patient 21

To identify disease-causing variants in Patient 21, whole genome sequencing was conducted following the acquisition of written informed consent for clinical and genomic research purposes. DNA was extracted from buccal swab of probands, and libraries were prepared using the TruSeq NanoDNA High Throughput Library Prep Kit (Illumina®). Sequencing performed on an Illumina platform using the 150 nt paired-end protocol achieving an average coverage depth of 30x for the nuclear genome and at least 1000x for the mitochondrial genome. Raw read alignment to reference genome GRCh38 and variant calling, including single nucleotide substitutions (SNVs), small insertions/deletions (Indels) and structural variants (SVs) with default parameters were performed using DRAGEN (version 4.2.4, Illumina). SNV and Indel variant annotation was performed by Geneyx (https://geneyx.com). Structural variants were annotated with ANNOTSV3.1 and inhouse structural variant databases to obtain allele frequencies. For the mitochondrial genome, variants with frequencies/heteroplasmy level ≥5% are detected. Genetic variants are described following the Human Genome Variation Society (HGVS) recommendations (www.hgvs.org). The selected variants were classified according to ACMG and ClinGen guidelines.

Cloning and plasmid constructs.

miRNAs against coding sequences (CDSs) for mouse *Adat3* (NM_001100606), *Adat2* (NM_025748.4), were generated using BLOCK-iT™ RNAi Designer (https://rnaidesigner.thermofisher.com/rnaiexpress/). Sense and antisense oligos (**Supplementary Table 5**) were annealed and the resulting duplex was subcloned in pCAGGs-mir30 (Addgene plasmid # 14758)[8] or NeuroD-miR30 vectors digested with XhoI and EcoRI. NeuroD-miR30 vector was generated by replacing the pCAGGs promoter in pCAGGSs-miR30 backbone with NeuroD promoter from the NeuroD-IRES-GFP plasmid.[9]

Wild-type (WT) mouse *Adat3 (*NCBI Reference Sequence NM_001100606) *and Adat2* (NM_025748.4 CDSs were amplified from E16.5 cortices using primers listed in **Supplementary Table 5** and cloned into pJET 1.2 blunt vector using CloneJet PCR Cloning kit. They were further subcloned into psiSTRIKE DCX-IRES-GFP (provided by J. Chelly (IGBMC, Strasbourg, France)) and pnThx[10] *(Adat3)*, pet16B (Novagen (EMD Millipore)) (*Adat2*), NeuroD-IRES-GFP[9] (*Adat2*), pCAGGs-IRES-GFP[11] (*Adat2, Adat3*) vectors by restriction-ligation. For bacterial expression, the m*Adat2* gene was inserted in the pnCS vector that does not code for any fusion tag. The m*Adat3* gene was inserted in the pnEA-HT3 vector, in frame with a 5′-sequence coding for an N-terminal histidine-tag, thioredoxin and a protease 3C cleavage site.[12] miR1-*Adat3* resistant constructs were obtained by site-directed mutagenesis using the primers indicated in **Supplementary Table 6** and subcloned into the psiSTRIKE DCX-IRES-GFP, and pCAGGs-IRES-GFP plasmids. Insensitivity of the vectors was validated by transfection of HEK239T cells together with the miRNA constructs. Mouse *Adat3* V128M, A180L and A180V variants were created from WT CDS by sequence- and ligation-independent cloning (SLIC) and subcloned into the psiSTRIKE DCX-iresGFP and pnThx[10] vectors. Mouse catalytically inactive Adat2 (E73A)[13] was generated from the WT *Adat2* CDSs respectively by site-directed mutagenesis using the primers listed in **Supplementary Table 6**. All the vectors used in this study were prepared using the EndoFree plasmid purification kit (Macherey Nagel).

Generation of Rabbit antibodies for mouse ADAT3 and ADAT2.

WT mouse mADAT3 and mADAT2 full length proteins were expressed by transformation of pnThx-*Adat3*, pet16B-*Adat2* vectors[10] into BL21 (DE3) Rosetta®(DE3) *E. coli* cells (Novagene). Bacterial cultures were grown in 2XLB media for 5-6 hours at 37ºC and 200rpm. Temperature was then decreased to 22ºC and

recombinant protein expression was induced by addition of 0.5 mM IPTG to the LB culture media that was grown O/N at 180rpm. Next day, Cultures were harvested, resuspended in resuspension buffer (200mM NaCl, 10mM Tris pH8) and sonicated on ice. The lysate was centrifuged and the supernatant was incubated with Talon Metal Affinity Resin (Clontech) for two hours at 4ºC. The resin was washed with resuspension buffer to get rid of the unbound proteins. Adat3 was eluted from the column by incubating the resin with 3C protease and Adat2 was eluted by by addition of 200mM NaCl, 10mM Tris, 250mM Imidazole (pH8) buffer. Eluted proteins were concentrated using Amicon® Ultra 15ml Centrifugal Filters (Merck) and loaded on HiLoad® 16/600 Superdex® columns (Akta Pure, Purification system) for affinity-based protein purification. Eluted fractions containing mouse ADAT3, ADAT2 proteins were respectively pooled and dialyzed against PBS overnight using 3.5K Slide-A-Lyzer™ G2 Dialysis Cassettes (ThermoFisherScientific).

300 µg of purified recombinant WT mouse ADAT3 and ADAT2 full length proteins were used for immunization of rabbits. One month later, 40 ml of blood was drawn every week four times and the serums were collected. Rabbits were boosted with 150 µg of peptide (in a 1:1 PBS/incomplete Freund adjuvant emulsion) and killed 12 days later, under anesthesia. Antibodies were purified from serum with SulfoLink-columns coupled to the immunogens (20325, Thermo Fischer Scientific) according to manufacturer's protocol and specificity validated by Western Blot **(Supplementary Fig. 1B)**.

Mice

All animal studies were conducted in accordance with French regulations (EU Directive 86/609 – French Act Rural Code R 214-87 to 126) and all procedures were approved by the local ethics committee and the Research Ministry (APAFIS#15691-201806271458609 and #4220-2016022318474293). Mice were bred at the IGBMC animal facility under controlled light/dark cycles, stable temperature (19°C) and humidity (50%) condition and were provided with food and water ad libitum. Timed-pregnant WT NMRI (Janvier-labs) and CD1 (Charles River Laboratories) females were used for *in utero* electroporation of the different constructs at embryonic day 14.5 (E14.5).

In utero electroporation

In utero electroporation (IUE) was performed as described previously.[14,15] Briefly, CD1 pregnant females were anesthetized with isoflurane (2L/min of oxygen; 4% isoflurane in the induction phase followed by two % isoflurane during surgery; Tem Sega). The uterine horns were exposed, and a lateral ventricle of each embryo was injected using pulled glass capillaries (Harvard apparatus, 1.0OD*0.58ID*100mmL) with Fast Green (1 µg/µl; Sigma) combined with different amounts of DNA constructs using a micro injector (Eppendorf Femto Jet). Plasmids were electroporated into the neuronal progenitors adjacent to the ventricle by 5 electric pulses (40V) for 50 ms at 950 ms intervals using electrodes (diameter 3 mm; Sonidel CUY650P3) and ECM-830 BTX square wave electroporator (VWR international). After electroporation, embryos were placed back in the abdominal cavity and the abdomen was sutured using surgical needle and thread. For E18.5 analysis, pregnant mice were sacrificed by cervical dislocation four days after surgery. For post-natal analysis, electroporated pups were sacrificed two days after birth (P2) by head sectioning. Conditions of IUE with plasmids and concentration used are summarized in **Supplementary Table 7.**

Mouse brain fixation, cutting and immunolabelling

E18.5 and P2 animals were sacrificed by head sectioning and brains were fixed in four % paraformaldehyde (PFA, Electron Microscopy Sciences) diluted in Phosphate buffered saline (PBS, HyClone) overnight at 4°C. WT E18.5 cryosections were prepared for immunolabeling as follows: after fixation, brains were rinsed and equilibrated in 20% sucrose in PBS overnight at 4°C, embedded in Tissue-Tek O.C.T. (Sakura), frozen on dry ice, cut coronally at the cryostat (18 µm thickness, Leica CM3050S) and maintained at -80°C until immunolabeling. For IUE analyses, vibratome section were prepared as follows: after fixation, brains were washed and embedded in a 4% low-melting agarose solution (Bio-Rad) and cut at a thickness of 60µm coronally using a vibrating-blade microtome (Leica VT1000S, Leica Microsystems). Sections were kept in PBS-azide 0.05% for short-term storage or in an antifreeze solution (30% Ethyleneglycol, 20% Glycerol, 30% DH2O, 20% PO_4 buffer) for long-term storage. For immunolabeling cryosections and vibratome sections were permeabilized and blocked with blocking solution (5% Normal Donkey Serum (NDS, Dominic Dutscher), 0.5% Triton-X-100 in PBS) for one hour at room temperature (RT). Sections were then incubated

with primary antibodies (see **Supplementary Table 7**) diluted in blocking solution overnight at 4°C and with secondary antibodies (see **Supplementary Table 7**) and DAPI (dilution 1/1000, 1mg/mL Sigma) diluted in PBS 0.1% Triton for one hour at RT. Slides were mounted using Aquapolymount mounting medium (Polysciences Inc).

Primary neuronal culture and immunolabeling

Cortices from WT CD1 mice at E15.5 were dissected in cold PBS supplemented with BSA (3 mg/mL), MgSO4 (1 mM, Sigma), and D-glucose (30 mM, Sigma). They were enzymatically dissociated in Neurobasal medium containing papain (20 U/mL, Worthington) and DNase I (100 µg/mL, Sigma) for 20 minutes at 37°C, washed five minutes with Neurobasal medium containing Ovomucoide (15 mg/mL, Worthington) and manually triturated in Optimem with 20mM D-glucose. 2 x 10^5 cells per well were plated in a 24-well plate previously coated overnight at 4°C with poly-D-lysine (1 mg/mL, Sigma). Cells were then either fixed two hours after plating or cultured in Neurobasal medium supplemented with B27, L-glutamine (2 mM) and penicillin-streptomycin (5 U/mL and 50 mg/mL, respectively) till DIV2 and fixed in 4% PFA and 4% sucrose in PBS for 15 minutes at RT. Cells were then blocked for 1 hour in 0,1% Triton X-100, 5% NDS in PBS and primary antibodies (see **Supplementary Table 8**) were added overnight at 4°C. Next day they were washed and incubated with secondary antibodies (see **Supplementary Table 8**) and DAPI (dilution 1/1000, 1mg/mL Sigma) for 1 hour at RT. Subsequently, they were mounted in Aquapolymount mounting medium (Polysciences Inc).

Cell culture and transfections

Human embryonic kidney 293T (HEK293T) cells were cultured in Dulbecco's modified Eagle's medium (DMEM, GIBCO) with 10% foetal calf serum (FCS), penicillin 100 U/mL and streptomycin 100 µg/mL. Mouse neuroblastoma N2A (ATCC) cells were cultured in DMEM (GIBCO) supplemented with 5% Fetal Calf Serum (FCS) and Gentamycin 40 µg/ml in a humidified atmosphere containing 5% CO2 at 37°C.

All the cells were incubated in a humidified atmosphere containing 5% CO2 at 37°C. Human lymphoblastoid cell lines (LCLs) were generated from blood samples of Patients 6 and 7 or has been previously described

in[1]. LCLs were cultured in RPMI 1640 medium containing 15% fetal bovine serum, 2 mM L-alanyl-L-glutamine (GlutaMAX; Gibco), and 1% penicillin-streptomycin.

For transfection, when cells reached 40-60% confluence, they were transfected using Lipofectamine 2000 (Invitrogen) according to the manufacturer's protocol. 48h post-transfection expression of transfected genes was assessed by RT-qPCR and western blot analysis. To assess miRNAs or shRNA knock-down efficacy and validate specificity of antibodies, HEK293T cells were transfected with 1 µg of pCAGGs-*Adat3*-IRES-GFP, pCAGGs-*Adat2*-IRES-GFP, and 3 µg of pCAGGs-miR30-scramble or pCAGGs-miR30-miRNA targeting *Adat3*, *Adat2*, respectively. To validate miRNA resistant vectors, HEK293T cells were transfected with 1 µg of either pCAGGs-*Adat3*-IRES-GFP (WT or miRNA resistant) together with 3 µg of pCAGGs-miR30-scramble or pCAGGs-miR30-miRNA targeting *Adat3*. For confirmation of expression of mutant vectors 1 µg of the respective psiSTRIKE DCX-IRES-GFP or NeuroD-IRES-GFP vectors were transfected in N2A cells.

RNA extraction, cDNA synthesis and RT–qPCR

Total RNA from brain tissues (NMRI) or cells was extracted using TRIzol reagent (Thermo Fischer Scientific). and submitted to DNAseI treatment (TurboDNAse, ThermoFisher). cDNA samples were synthetized with SuperScript IV Reverse Transcriptase (Invitrogen) and quantitative RT-PCR (qRT-PCR) was done with amplified cDNA and SYBR Green Master Mix (Roche) together with 0.1 µM of forward and reverse primers using a Lightcycler® 480 (Roche). RT-qPCRs on WT NMRI mouse cortices from E12.5-E18.5 embryos, human LCLs and HEK293T cells transfected with different constructs were carried using the primers listed in **Supplementary Table 6**.

Protein extraction and western blot

Proteins from mouse cortices (E12.5 to P2, NMRI), transfected cells (HEK 293T, N2A) and LCLs were extracted as follows: cells or tissue were lysed in RIPA buffer (50 mM Tris pH 8.0, 150 mM NaCl, 5 mM EDTA pH 8.0, 1% Triton X-100, 0.5% sodium deoxycholate, 0.1% SDS) supplemented with EDTA-free protease inhibitors (cOmplete™, Roche) for 30 min, then cells debris were removed by high speed centrifugation at 4°C for 25 min. Protein concentration was measured by spectrophotometry using Bio-Rad

Bradford protein assay reagent. Samples were denatured at 95°C for 10 min in Laemmli buffer (Bio-Rad) with 2% β-mercaptoethanol, then resolved by SDS–PAGE and transferred onto nitrocellulose membranes. Membranes were blocked in 5% milk in PBS buffer with 0.1% Tween (PBS-T) and incubated overnight at 4°C with the appropriate primary antibody in blocking solution. Membranes were washed three times in PBS-T, incubated at room temperature for one hour with HRP-coupled secondary antibodies at 1:10,000 dilution in PBS-T, followed by three times PBS-T washes. Visualization was performed by quantitative chemiluminescence using SuperSignal West Pico PLUS Chemiluminescent Substrate (Sigma). Signal intensity was quantified using ImageQuant LAS 600 (GE Healthcare). Primary and secondary coupled HRP antibodies used for western blot are described in **Supplementary Table 8**. All immunoblot experiments consisted of at least three independent replicates.

Small-scale expression tests of mADAT2/ADAT3

For small-scale expression tests, pnCS-mADAT2 WT and pnEA-HT3-mADAT3 WT and mutants were expressed alone or co-expressed in *Escherichia coli* BL21(DE3) cells. Transformed cells were cultivated at 37°C for 6 hours in 24 deep well plates harbouring 4 mL of 2xLB Broth medium and the required antibiotics in each well. Induction was then performed overnight at 22°C by adding a final concentration of 0.7 mM of isopropyl-1-thio-β-D-galactopyranoside (IPTG) in the presence of 100 μM of $Zn(SO_4)_2$. Cells were harvested and resuspended in a buffer containing 10 mM Tris-HCl pH 8.0 and 200 mM NaCl. For total protein expression analysis, 100 mL of the resuspended cells were mixed with 10 μL of Laemmli buffer and heated for 10 minutes at 95°C prior to analysis by SDS-PAGE followed by Coomassie staining. The rest of the resuspended cells were lysed and centrifuged at 4 000 rpm for 30 m at 4°C, and the supernatants incubated with TALON Metal Affinity Resin (Clonetech) for two hours at 4°C, then centrifuged again and washed two times. For soluble protein analysis, the TALON resin was mixed with 20 μL of Laemmli buffer and heated for 10 minutes at 95°C prior to analysis by SDS-PAGE.

Structure of the ADAT complex

Large-scale overproduction and purification of mADAT2/ADAT3

mADAT2/ADAT3 WT and mutants were produced by co-expression in *E. coli* BL21(DE3) cells. Cultures were cultivated in 2xLB Broth medium at 37°C for 6 hours. Induction was then performed at 22°C overnight by adding final concentration of 0.7 mM of IPTG and 100 µM of $Zn(SO_4)_2$. Cells were harvested, resuspended and lysed in a buffer containing 10 mM Tris-HCl pH 8.0 and 200 mM NaCl and centrifuged at 17 500 rpm for one hour at 4°C. The supernatant was incubated with TALON Metal Affinity Resin (Clonetech). To release the his-tagged complex from the TALON resin, the sample was treated with 3C protease overnight at 4°C. The next day, ion exchange chromatography was performed with a HiTrap Q HP column (GE Healthcare) using a gradient of NaCl from 50 mM to 1 M NaCl to remove bound nucleic acids. The sample was then further purified by size exclusion chromatography in 10 mM Tris HCl pH 8.0, 200 mM NaCl and 0.5 mM TCEP on a 16/600 Superdex 200 gel filtration column (GE Healthcare). The recombinant complexes were used for crystallization assays and enzymatic deamination assays.

Protein crystallization

For crystallization, the A180V and A180L mutant ADAT complexes at 12 mg/ml were mixed with an equal volume of reservoir reagent and crystallized using the sitting drop vapor diffusion technique at 20°C. All crystals grew within one week. Crystals could only be obtained for the A180V mutant ADAT complex using a crystallization solution containing 0.1 M Bis-Tris-Propane pH 6.5, 20.5% PEG 3350 and 0.2 M NaBr.

Data collection, structure determination, model building and refinement

For data collection, the crystals were frozen in liquid nitrogen after their short transfer into a cryo-protectant solution composed of their crystallization conditions added with either 20% glycerol or 20% PEG200. Data collection was performed under cryogenic conditions on beamline PXIII at the Swiss Light Source synchrotron (SLS, Switzerland) using a 1 Å wavelength. Data sets collected were processed with XDS.[16] Structure determination was made by molecular replacement using our previous structure of the WT mADAT2/ADAT3 complex (PDB entry #7NZ8) and the structure refined by several cycles of manual building

using Coot[17] and automated refinement using Phenix.[18] The final model was validated using tools provided in Coot and Molprobity.[19]

Enzymatic deamination assays

tRNA production was done as follow: The mouse $tRNA^{Arg}$(ACG) gene used was synthesized using two complementary oligonucleotides (Merk) comprising the tRNA gene sequence (underlined), a T7 RNA polymerase promoter (bold), a BstNI site (italics), and two restriction sites, HindIII and BamHI (bold underline):

5’-**AGCTTGAATTGTAATACGACTCACTATA**GGGCCAGTGGCGCAATGGATAACGCGTCTGACTAC

GGATCAGAAGATTCTAGGTTCGACTCCTAGCTGGCTCG*CCAGG***G**-3’ and

5’-

GATCC*CCTGG*CGAGCCAGCTAGGAGTCGAACCTAGAATCTTCTGATCCGTAGTCAGACGCGTTATC

CATTGCGCCACTGGCCC**TATAGTGAGTCGTATTACAATTCA**-3’.

For the oligonucleotide hybridization, 4 µg of each oligonucleotide were first incubated separately at 37°C for 45 min in the presence of 4 µl of T4 DNA Ligase buffer from the T4 DNA ligase kit (Thermo Scientific™ Cat.#EL0011) and 1 µl of T4 Polynucleotide Kinase (10U/µl) from the T4 Polynucleotide Kinase kit (Thermo Scientific™ Cat.#EK0031) in a total volume of 25 µl. The two primer solutions were mixed, incubated at 100°C for one minute, then at 70°C for 25 minutes in a water bath, and let to cool down to 20°C for two hours in the switch-off water bath. The hybridized oligos were inserted in the HindIII and BamHI-linearized pUC19 vector using the T4 DNA ligase kit (Thermo Scientific™ Cat.#EL0011) according to the manufacturer's instructions.

The tRNA was synthesized from the BstNI-digested DNA by *in vitro* transcription using recombinant T7 RNA polymerase (doi.org/10.1042/BJ20121211). After transcription, the sample was treated with RQ1 RNase-Free DNase (Promega), and the RNA transcript was phenol-extracted and precipitated. Pelleted tRNA was dissolved in water and loaded on 7 M Urea-15 % acrylamide, and 1 X TBE gel. After methylene blue staining, gel slices containing the tRNA transcript were cut from the gel. The tRNA was eluted overnight

at room temperature in 0.5 M ammonium acetate, 10 mM magnesium acetate, 0.1 mM EDTA and 0.1% SDS. After phenol extraction, tRNA was ethanol precipitated and finally recovered in water. The concentration was determined by absorbance measurements. The tRNA was then used for enzymatic deamination assays.

Deamination assays were done in deamination buffer (10 mM Tris-HCl pH 8.0, 100 mM NaCl, 1 mM $MgCl_2$, 2 mM dithiothreitol (DTT)) using 2 µM of tRNA transcript and 5.6, 0.56 or 0.056 µM of purified enzyme complex (tRNA:protein ratios of approximately 1:3, 1:0.3 and 1:0.003) in a final volume of 10 µL. The reaction was initiated when the purified enzyme complex was added to the reaction mixture and immediately incubated at 37°C for the indicated time (10 min). The reaction was immediately stopped by phenol-chloroform extraction. The supernatant was precipitated, and the pellet containing the tRNA transcript was dissolved in 20 µl of water. The cDNA was synthesized using the SuperScript™ IV Reverse Transcriptase (Invitrogen Cat.# 18090010) according to the manufacturer's instructions with 2 µl of the tRNA transcript solution and 0.1 µM of gene-specific complementary primer. The tRNAs were then amplified using the GoTaq® G2 Flexi (Promega Cat.# M7801) according to the manufacturer's instructions with 5 µl of cDNA, 2.5 mM of $MgCl_2$, and 1 µM of gene-specific primers. As PCR fragments were too small for direct sequencing, they were ligated using the pGEM®-T Easy Vector System (Promega Cat.# A1360) according to the manufacturer's instructions. The ligated PCR products were then reamplified using vector-specific and tRNA-specific primers. The resulting PCR fragments were precipitated, redissolved, and directly sequenced with the vector-specific primer. The A-to-I deamination reaction was visualized by the presence of a guanosine peak at the adenosine peak position since reverse transcriptase incorporates a cytosine instead of an inosine. The deamination rate was quantified using the peak area measurement in the Raw Data section of MacVector software (version 18.6.1). The percentage of inosine was calculated using the A and G area data sets. The forward and reverse primers specific to $tRNA^{Arg}$ and used for RT-PCR and PCR were as follows: 5'-GGGCCAGTGGCGCAATGGA-3' and 5'TGGCGAGCCAGCTAGGAGT-3'. The forward primer for the pGEM®-T Easy Vector was as follows: 5'-GTAAAACGACGGCCAG-3'.

tRNA-seq

tRNA-seq library construction

LCLs derived from patients and healthy controls were lysed in lithium dodecyl sulfate (LiDS)/LET buffer (5% LiDS in 20 mM Tris, 100 mM LiCl, 2 mM EDTA, 5 mM dithiothreitol (DTT) pH 7.4 and 100 μg ml^{-1} proteinase K). The lysates were incubated at 60 °C for 10 min, pushed ten times through a 1 ml syringe with a 26 G needle and mixed by vortexing. Two volumes of cold acid phenol (pH 4.3), 1/10 volume 1-bromo-3-chloropropane and 50 μg glycogen (Thermo Fisher Scientific, AM9510) were added to the lysate and samples were mixed vigorously by vortexing, followed by centrifugation at 10,000*g* at 4 °C. The aqueous phase was transferred to a new tube and the phenol and 1-bromo-3-chloropropane extraction was repeated. RNA was then precipitated from the aqueous phase by the addition of three volumes of 100% ethanol and incubation at −20 °C for 30 min. The pellets were washed with 80% ethanol, air-dried and resuspended in RNase-free water. The RNA concentration was measured using a Nanodrop system and the samples were stored at −80 °C. The tRNASeq libraries were prepared using the mim-tRNAseq workflow.[20-22] Briefly, total RNA from two biological replicates for each LCL line was mixed with synthetic *Escherichia coli* tRNA-Lys-UUU-CCA and *E. coli* tRNA-Lys-UUU-CC at a 3:1 ratio, followed by dephosphorylation with T4 PNK (NEB, M0201S) and ethanol precipitation. The RNA samples were resolved on denaturing 10% polyacrylamide, 7 M urea and 1×TBE gels. RNA of 60-100 nt was excised and eluted from the gel followed by ethanol precipitation. The gel-purified tRNA was then ligated to pre-adenylated, barcoded 3′-adaptors [20] in 1×T4 RNA ligase buffer, 25% PEG-8000, 20 U Superase In (Thermo Fisher Scientific, AM2696) and 1 μl T4 RNA ligase 2, truncated KQ (NEB, M0373S). The mix was incubated for 3 h at 25 °C and the ligation products were purified by size selection on a 10% polyacrylamide, 7 M urea and 1×TBE gel. Adaptor-ligated tRNA (100 ng) was annealed with 1 μl of 1.25 μM RT primer (5′ - pRNAGATCGGAAGAGCGTCGTGTAGGGAAAGAG/iSp18/GTGACTGGAGTTCAGACGTGTGCTC-3′, where iSp18 is a 18-atom hexa-ethyleneglycol spacer) at 82 °C for 2 min, followed by incubation at 25 °C for 5 min. Reverse transcription was performed with 500 nM TGIRT (InGex, TGIRT50) in 50 mM Tris–HCl pH 8.3, 75 mM KCl, 3 mM $MgCl_2$, 5 mM DTT (from a freshly prepared 100 mM stock), 1.25 mM dNTPs and 20 U Superase In at 42 °C for 16 h. After reverse transcription, NaOH was added to a final concentration of

0.1 M and the RNA was hydrolyzed by incubating the samples for 5 min at 90 °C. Complementary DNA products were separated from unextended primer on a 10% polyacrylamide, 7 M urea and 1×TBE gel. Regions corresponding to cDNAs that were >10 nt longer than the RT primer were excised after SYBR Gold staining. Gel-purified and ethanol-precipitated cDNA was incubated for three hours at 60 °C with CircLigase ssDNA ligase (Lucigen) in 1×reaction buffer supplemented with 1 mM ATP, 50 mM $MgCl_2$ and 1 M betaine. Following enzyme inactivation for 10 min at 80 °C, one-fifth of the circularized cDNA was used directly for library construction PCR with a common forward (5′-AATGATACGGCGACCACCGAGATCTACACTCTTTCCCTACACGACGCT∗C-3′) and unique indexed reverse primers (5′-CAAGCAGAAGACGGCATACGAGATNNNNNNGTGACTGGAGTTCAGACGTGT∗G-3′; NNNNNN, the reverse complement of an Illumina index sequence; asterisk, phosphorothioate bond) with KAPA HiFi DNA polymerase (Roche) in 1×GC buffer with initial denaturation at 95 °C for three minutes, followed by five cycles of 98 °C for 20 s, 62 °C for 30 s and 72 °C for 30 s at a ramp rate of 3 °C s^{-1}. The PCR products were purified using a DNA Clean and Concentrator 5 kit (Zymo Research), quantified with a Qubit dsDNA HS kit (Thermo Fisher Scientific, Q32851) and sequenced for 150 cycles on an Illumina NextSeq 500 platform, generating >2.5 × 10^6 reads per library.

Analysis of tRNASeq data

The tRNASeq data was analyzed according to the mim-tRNAseq computational workflow.[20-22] Demultiplexing and 3′ sequencing adaptor removal was performed using cutadapt v3.5. Indels were disallowed (--no-indels) and both read ends were quality trimmed with a quality score of 30 (-q 30,30). As sequencing was performed with more cycles than the length of any sequenced fragment, all reads were expected to contain adaptors and only trimmed reads were retained with --trimmed-only. The reads were further trimmed to remove the two 5′-RN nucleotides introduced by circularization from the RT primer with -u 2. In both processing steps, reads <10 nt were discarded using -m 10. Analysis of tRNA expression and modification was performed with v1.3.8 of the mim-tRNAseq computational package (https://mim-trnaseq.readthedocs.io/en/latest/index.html).[21] Briefly, the full set of 619 predicted tRNA genes for the hg38 human genome assembly were downloaded from GtRNAdb[23] and the 22 mitochondrially encoded human tRNA genes were fetched from mitotRNAdb[24]. After intron removal and the addition of 5′-G (for tRNA-His)

and 3′-CCA (for nuclear-encoded transcripts), a curated set of 599 nuclear-encoded tRNA sequences (excluding tRNAs with non-canonical secondary structure alignments or undetermined anticodons) and 22 mitochondrially encoded tRNA sequences was compiled as an alignment reference (--species Hsap). The reads were aligned to this reference with a cluster ID of 0.95, maximum mismatch tolerance at a number of nucleotides equal to 7.5% read length for the first alignment round and 5% read length for realignment, a deconvolution coverage ratio of 0.4 at mismatch sites to allow accurate cluster deconvolution and a minimum coverage threshold of 0.05% total reads per transcript for low coverage transcript filtering. In addition, DESeq2 was run on tRNA transcripts with single-transcript resolution by first removing those still in clusters from the counts table (evidenced by the presence of multiple transcripts in the name, separated by '/') and repeating DESeq2 analysis on these. Isotype counts, generated by aggregating anticodon counts for the same tRNA isotype were also generated, and DESeq2 was additionally run on this count data.

Inosine 34 proportions were obtained from the mismatch analysis integrated in the mimtRNA-seq pipeline. For each sample, mismatch values for the canonical position 34 matching the nucleotide G were obtained and averaged across replicates as follows. For the anticodon-level analysis, the mean across all isodecoders of the same isotype weighted by their coverage was calculated and then averaged across replicates. For the isodecoder-level analysis, the mean across replicates was weighted by their total coverage. For all other modifications, given the absent of a clear mis-read designed nucleotide, the misincorporation rate at the 7 canonical positions resolved from mimtRNAseq for the ADAT-target genes (position 9 = m^1G or m^1A, position 20 = acp^3U, position 26 = $m^{2,2}G$, position 32 = m^3C, position 37 = yW or m^1I, position 58 = m^1A) was calculated for each condition as for I34. tRNAs never modified in the controls in such positions (misincorporation proportion < 1%) were removed.

Codon enrichment analysis

For evaluating the codon enrichment in human genes associated to neuronal migration, we compared codon frequencies in neuronal migration associated-genes versus either the frequencies obtained from the human brain cortex Codon and Codon Pair Usage Tables (CoCoPUTs)[25] (https://dnahive.fda.gov/dna.cgi?cmd=codon_usage&id=537&mode=cocoputs) or non-neuronal migration associated-genes.

To define the genes associated to neuronal migration or non-neuronal migration, we relied on the gene ontology (GO) resource (https://geneontology.org/). We first obtained all available GO entries using GO.db v.3.19.1 in R and defined as neuronal-migration-associated all those entries that contained the words "migration" and "neuro". Fifty-six entries were retained. All genes associated to these ontologies were obtained from the R annotation data package org.Hs.eg.db v.3.19.1. This process yielded a total of 202 genes. We then used the R package biomartr v.1.0.7[26] to retrieve all their available coding sequences from the database Ensembl (193 genes total). For each gene only sequences starting with a codon "ATG" and showing triplets periodicity were retained. In case of multiple transcripts available per gene, the longest one was kept. Similarly, all entries containing the word "migration" but not the word "neuro" (331 GO entries) were used as non-neuronal migration and their sequence was retrieved as explained above (1316 final sequences).

For each gene we calculated the abundance of each codon and the total abundance for each gene group was calculated as the sum, excluding the starting ATGs. Finally, overall frequency of each codon was calculated across the summed abundances. For each comparison, the deltas between the codon frequency of neuronal migration genes and their comparison (either the CoCoPUT frequency table or the non-neuronal migration genes) were calculated. All codons were then assigned their translating anticodon by matching their sequence and taking into consideration the existence of wobbling for those codons whose matching anticodon is missing. A codon was defined as DEG-tRNA if its translation is done by a tRNA-anticodon found dysregulated in our anticodon-level mimtRNAseq results. Differences between DEG-tRNAs codons and non-DEG-tRNA codons were tested by a Wilcoxon rank sum test in R given their deviance from normality.

Relative synonymous codon usage (RSCU) was calculated for all the codons within the 8 ADAT-target families (Ala, Arg, Ile, Leu, Pro, Ser, Thr, and Val) across all genes associated to neuronal migration (see above) or the whole genome. Coding sequences for either these genes or the whole human genome were obtained as explained above. For each gene, we then calculated RSCU across all genes using the function *uco* integrated in the R package seqinr v.4.2-36.[27] Differences in RSCU between the two groups were pair-wise tested using the Dunn's test in rstatix v.0.7.2 (https://rpkgs.datanovia.com/rstatix/).

Image acquisition and analysis

Images for primary neuronal culture and expression pattern analyses were acquired using a TCS SP8 UV (Leica microsystems) using a 63x OIL HC PL APO CS2 and 20x IMM, HC PL APO CS2 objectives respectively and images for neuronal migration and expression pattern analyses were acquired using a TCS SP8 X (Leica microsystems) confocal microscope using a 20x DRY HC PL APO CS2 objective. For all experiments, a Z-stack of 1,50 μm was acquired. The image size was 512x512 for neuronal migration analysis and 1024x1024 for primary neuronal culture and expression analysis. Image analysis was done using ImageJ software (NIH). Cell counting was performed in two to four different brain sections of at least three different embryos or pups per condition. Only similarly electroporated regions were considered for further analysis. Cortical areas (upper cortical plate, lower cortical plate, intermediate zone, subventricular zone/ventricular zone) were delimited based on cell density (nuclei count with DAPI staining) using equivalent sized boxes. Number of GFP-positive cells was determined in each cortical area to establish the percentage of positive cells. All the experiments were done in at least three independent replicates.

Statistics

All statistics analyses were performed using GraphPad Prism 6 (GraphPad) and are represented as mean +/- S.E.M. The level of significance was set at $P < 0.05$ in all the statistical tests. All statistical tests used and n size numbers are shown in the figure legends and statistical details are reported in **Supplementary Table 9**. Correlation analysis was performed with R v. 4.4.0 (within R studio) and its package stats. Analysis of IUE experiments and neuroanatomical characterization of mouse brains were performed blinded. Normality was checked using Shapiro-Wilk or KS normality test depending on the sample size, when the data was big enough. In case normality was violated, non-parametric tests were used.

Supplementary Table 2: Blast analysis of the miRNAs used in this work.

microRNA Sequence	Target Name	Sequence ID	Identity	Gap	Coverage	Strand
Adat3 miR1 AGCATAGGCCAGTATCAGCTC	Adat3	NM_001100606.1	21/21	0/21	100%	Plus/Minus
	Macf1 transcript variant 1	NM_001199136.2	15/15	0/15	71%	Plus/Plus
	Macf1 transcript variant 2	NM_001199136.2	15/15	0/15	71%	Plus/Plus
	Sorcs3	NM_025696.3	17/18	1/18	86%	Plus/Plus
Adat3 miR2 ATAGCCAGTGCACACATAGGG	Adat3	NM_001100606.1	21/21	0/21	100%	Minus/Plus
Adat2 miR1 TCTGACAGCCATAGACGACC	Adat2	NM_025748.4	21/21	0/21	100%	Minus/Plus
	Rbp1	NM_011254.5	19/20	1/20	100%	Plus/Plus
	Dad1 transcript variant 2	NM_010015.4	15/15	0/15	71%	Plus/Minus
Adat2 miR2 TCAGCAGAGGCAATGTTTAGG	Adat2	NM_025748.4	21/21	0/21	100%	Minus/Plus
	Thbs2	NM_011581.3	16/16	0/16	76%	Plus/Plus

Supplementary Table 4: Crystallographic table.

**Data collection***	**mADAT A180V**
Space group	P 2 2_1 2_1
Cell dimensions	
a, b, c (Å)	51.90, 106.40, 129.20
α, β, γ (°)	90.0, 90.0, 90.0
Resolution (Å)	50. – 2.90 (3.08 – 2.90)
Rsym or Rmerge	35.5 (302.5)
I / σI	8.13 (1.12)
Completeness (%)	99.7 (99.8)
Redundancy	7.8 (8.1)
CC(1/2) (%)	99.1 (38.3)
**Refinement**	
Resolution (Å)	49.19 – 2.90
No. reflections	16383
Rwork / Rfree	0.195 / 0.245
Number of atoms	
Protein	3391
Ions	4
B-factors	
Protein	89.04
Ions	90.24
R.m.s. deviations	
Bond lengths (Å)	0.010
Bond angles (º)	1.129

Supplementary Table 6: List of sense and antisense oligos used in this work.

**miRNA and shRNA oligos**	
Scramble sense	5'TCGAGaaggtatattgctgttgacagtgagcgATCTCGCTTGGGCGAGAGTAAGtagtgaagccacagatgtaCTTACTCTCGCCCAAGCGAGAGtgcctactgcctcgG 3'
Scramble antisense	5'AATTCcgaggcagtaggcaCTCTCGCTTGGGCGAGAGTAAGtacatctgtggcttcactaCTTACTCTCGCCCAAGCGAGATcgctcactgtcaacagcaatataccttC 3'
Adat3 miR1 sense	5'TCGAGaaggtatattgctgttgacagtgagcgGAGCTGATACTGGCCTATGCTtagtgaagccacagatgtaAGCATAGGCCAGTATCAGCTC tgcctactgcctcgG 3'
Adat3 miR1 antisense	5'AATTCcgaggcagtaggcaGAGCTGATACTGGCCTATGCTtacatctgtggcttcactaAGCATAGGCCAGTATCAGCTCcgctcactgtcaacagcaatataccttC 3'
Adat3 miR2 sense	5'TCGAGaaggtatattgctgttgacagtgagcgCCCTATGTGTGCACTGGCTATtagtgaagccacagatgtaATAGCCAGTGCACACATAGGGtgcctactgcctcgG 3'
Adat3 miR2 antisense	5'AATTCcgaggcagtaggcaCCCTATGTGTGCACTGGCTATtacatctgtggcttcactaATAGCCAGTGCACACATAGGGcgctcactgtcaacagcaatataccttC 3'
Adat2 miR1 sense	5'TCGAGaaggtatattgctgttgacagtgagcgTGGTCGTCTATGGCTGTCAGAtagtgaagccacagatgtaTCTGACAGCCATAGACGACCAtgcctactgcctcgG 3'
Adat2 miR1 antisense	5'AATTCcgaggcagtaggcaTGGTCGTCTATGGCTGTCAGAtacatctgtggcttcactaTCTGACAGCCATAGACGACCAcgctcactgtcaacagcaatataccttC 3'
Adat2 miR2 sense	5'TCGAGaaggtatattgctgttgacagtgagcgCCTAAACATTGCCTCTGCTGAtagtgaagccacagatgtaTCAGCAGAGGCAATGTTTAGGtgcctactgcctcgG 3'
Adat2 miR2 antisense	5'AATTCcgaggcagtaggcaCCTAAACATTGCCTCTGCTGAtacatctgtggcttcactaTCAGCAGAGGCAATGTTTAGGcgctcactgtcaacagcaatataccttC 3'

***Cloning of Adat3, Adat2, Wdr4 and Mett1 CDSs* from mouse cortices**		
	**Forward sequence**	**Reverse sequence**

Adat3	5’ATGCAGCCCACCTCAGGCT3’	5’CTATGGGTCGGGGTCCAGCT3’
Adat2	5’ATGGAGGAGAAGGTGGAGTCCAC3’	5’TCAGGATTTCTGACAATCCTTTTTCCG3’

**Generation of microRNA resistant vectors**		
	**Forward sequence**	**Reverse sequence (mutagenic)**
Adat3 miR1 resistant	5’AAGCGCCAGACGTCCCGCCTC3’	5’GTCCAGGACAGGTGCAGCGTAAGCGGTATCAGCTCCAC3’

Cloning of gene variants		
	**Forward sequence**	**Reverse sequence**
Adat3 p.V128M	5’ CCTGGTGCCTATGCCTGCCC 3’	5’ AAAGGTGTGCCCAGGCCACGT 3’
Adat3 p.A180V	5’CCCACATGGAACGGGTGGTATGTGCGGCCCAG3’	5’CGCACATACCACCCGTTCCATGTGGGTTTG3’
Adat3 p.A180L	5’CCCACATGGAACGGTTGGTATGTGCGGCCCAG3’	5’CGCACATACCAACCGTTCCATGTGGGTTTG3’
Adat2 p.E73A	5’ATCAGGTCCTAGACTGGTGTCATCAGC 3’	5’ CAATGGCCACCATGGCAGCATG 3’

**RT-qPCR**		
	**Forward sequence**	**Reverse sequence**
Adat3	5’CTCAGTCGAGCCCCGTTG3’	5’CGGCTCTTGCTCCTCACTTT3’
Adat2	5’GCGCCTTATGAAAATCCCGC3’	5’AGGGATGCACTGAAACGGTC3’
Gapdh	5’GCACAGTCAAGGCCGAGAAT3’	5’GCCTTCTCCATGGTGGTGAA3’
HPRT1	5’AGGCGAACCTCTCGGCTTTC3’	5’TCATCATCACTAATCACGACGCC3’

Supplementary Table 7: Condition of *in utero* electroporation used in this work.

**Ubiquitous Knock-down experiments**	
**Plasmid**	**Amount**
NeuroD-IRES-GFP	1 µg/µl
pCAGGs-miR30-miRNA or pCAGGs-miR30-scramble	3 µg/µl
**Neuron-specific Knock-down experiments**	
**Plasmid**	**Amount**
NeuroD-IRES-GFP	1 µg/µl
NeuroD-miR30-miRNA or NeuroD-miR30-scramble	3 µg/µl
**Neuron-specific Knock-down rescue experiments (*Adat3*)**	
**Plasmid**	**Amount**
NeuroD-IRES-GFP	0.75 µg/µl
NeuroD-IRES-GFP or NeuroD-*Adat2*-IRES-GFP (WT or C.I)	0.5 µg/µl
NeuroD-miR30-miRNA or NeuroD-miR30-scramble	3 µg/µl
psiSTRIKE DCX-*Adat3* (WT or mutant) miRNA insensitive IRES-GF or psiSTRIKE DCX-IRES-GFP	0.75 µg/µl

Supplementary Table 8: List of primary and secondary antibodies used in this work.

Primary antibody	Host	Dilution	Antigen retrieval (IHC)	Used for	Provenance	Reference
Actin coupled HRP	Mouse	1/100 000	∅	WB	Sigma-Aldrich	A3854
GFP	Chicken	1/500	∅	IHC	Abcam	GFP-1020
Tbr2	Rat	1/250	∅	IHC	EBiosciences	14-4875-80
β-III-TUB	Mouse	1/200	∅	IHC	Eurogentec	MMS-435P-0100
α-Tubulin	Mouse	1/500	∅	IHC, WB	Merck	024M4767V
Adat3	Rabbit	1/500	2N HCl	IHC, WB	Homemade	∅
ADAT3	Rabbit	1/350	∅	WB	Sigma	HPA058899
Adat2	Rabbit	1/500	2N HCl	IHC, WB	Homemade	∅

Secondary antibody	Dilution	Used for	Provenance	Reference
Goat-mouse-HRP	1/10 000	WB	ThermoFisher Sc.	G-21040
Goat-rabbit-HRP	1/10 000	WB	ThermoFisher Sc.	G-21234
Goat-chicken-488	1/1000	IF	ThermoFisher Sc.	A-11039
Donkey-mouse-488	1/1000	IF	ThermoFisher Sc.	A-21202
Donkey-rat-488	1/1000	IF	ThermoFisher Sc.	A-21208
Donkey-rabbit-488	1/1000	IF	ThermoFisher Sc.	R-37118

Donkey-rabbit-555	1/1000	IF	ThermoFisher Sc.	A-31572
Donkey-mouse-555	1/1000	IF	ThermoFisher Sc.	A-31570
Donkey-mouse 647	1/1000	IF	ThermoFisher Sc.	A-31571

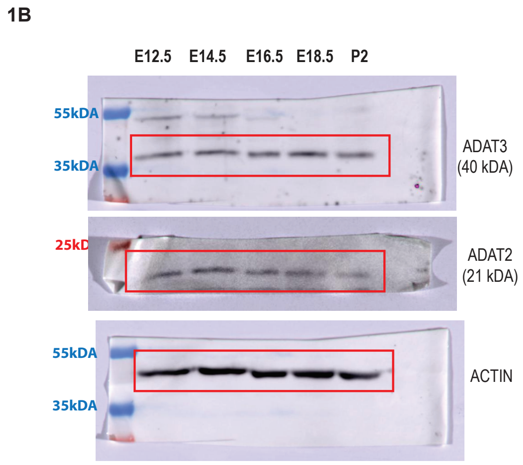
1B
E12.5 E14.5 E16.5 E18.5 P2
55kDA
35kDA
ADAT3
(40 kDA)
25kD
ADAT2
(21 kDA)
55kDA
35kDA
ACTIN

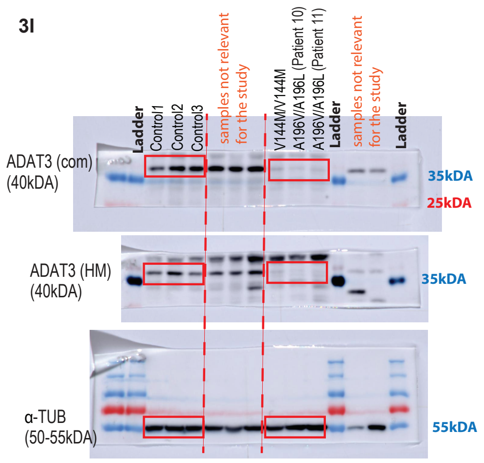
3I
Ladder
Control1
Control2
Control3
samples not relevant for the study
V144M/V144M
A196V/A196L (Patient 10)
A196V/A196L (Patient 11)
Ladder
samples not relevant for the study
Ladder
ADAT3 (com)
(40kDA)
35kDA
25kDA
ADAT3 (HM)
(40kDA)
35kDA
α-TUB
(50-55kDA)
55kDA

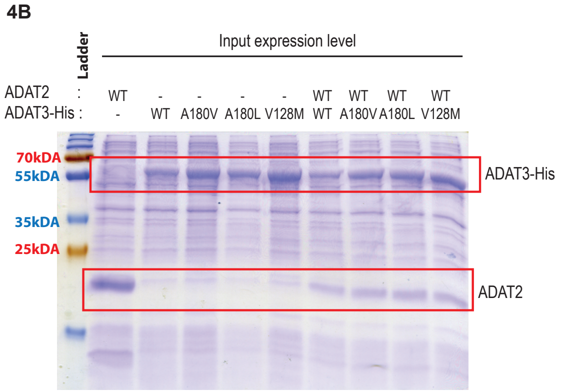
4B
Ladder
Input expression level
ADAT2 :
ADAT3-His :
WT -
- WT
- A180V
- A180L
- V128M
WT WT
WT A180V
WT A180L
WT V128M
70kDA
55kDA
35kDA
25kDA
ADAT3-His
ADAT2

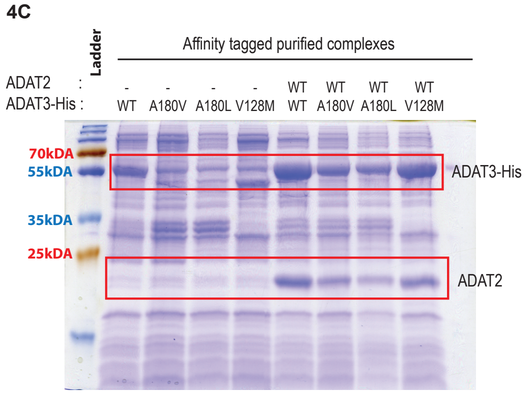
4C
Ladder
Affinity tagged purified complexes
ADAT2 :
ADAT3-His :
- WT
- A180V
- A180L
- V128M
WT WT
WT A180V
WT A180L
WT V128M
70kDA
55kDA
35kDA
25kDA
ADAT3-His
ADAT2

Supp 1B

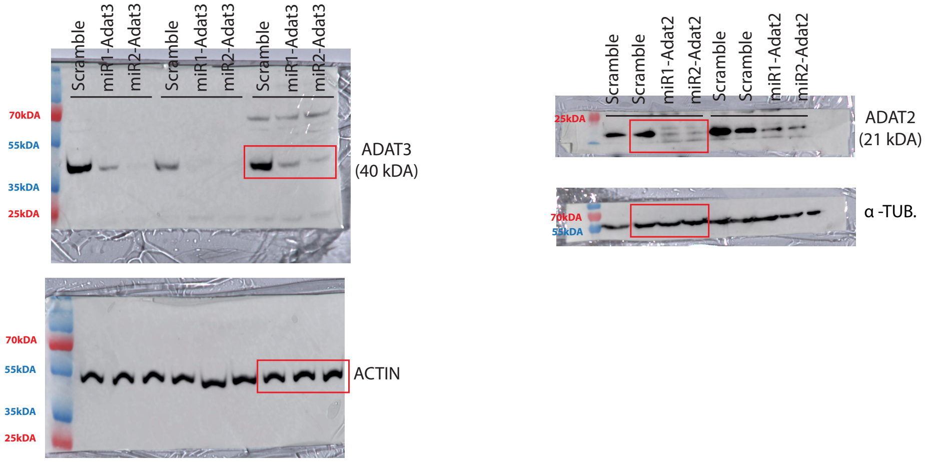

Supp 1F

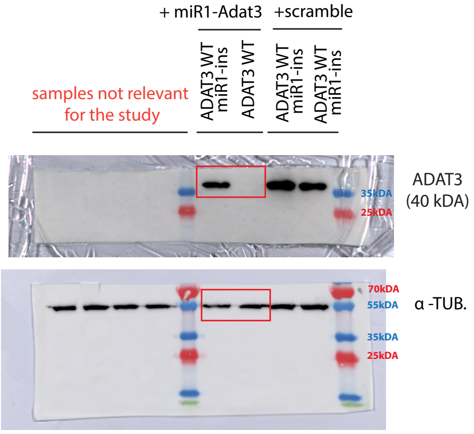

Supp 1I

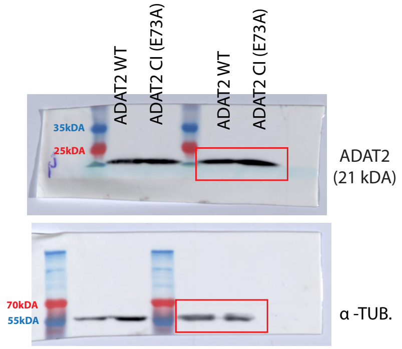

Supp 5A

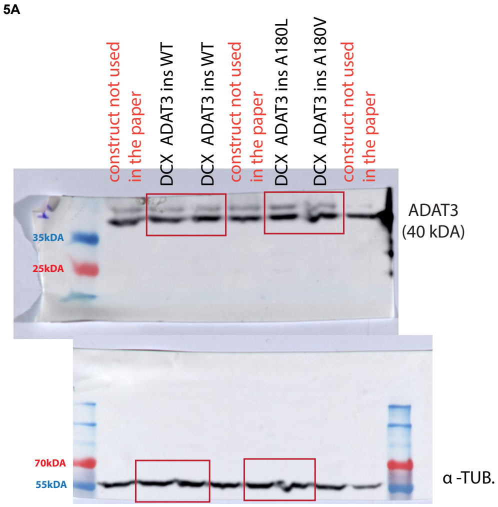

1. Ramos J, Han L, Li Y*, et al*. Formation of tRNA Wobble Inosine in Humans Is Disrupted by a Millennia-Old Mutation Causing Intellectual Disability. *Mol Cell Biol*. Oct 1 2019;39(19)doi:10.1128/MCB.00203-19

2. Thomas E, Lewis AM, Yang Y, Chanprasert S, Potocki L, Scott DA. Novel Missense Variants in ADAT3 as a Cause of Syndromic Intellectual Disability. *J Pediatr Genet*. Dec 2019;8(4):244-251. doi:10.1055/s-0039-1693151

3. Chopra M, Gable DL, Love-Nichols J*, et al*. Mendelian etiologies identified with whole exome sequencing in cerebral palsy. *Ann Clin Transl Neurol*. Feb 2022;9(2):193-205. doi:10.1002/acn3.51506

4. Li H, Durbin R. Fast and accurate short read alignment with Burrows-Wheeler transform. *Bioinformatics*. Jul 15 2009;25(14):1754-60. doi:10.1093/bioinformatics/btp324

5. McKenna A, Hanna M, Banks E*, et al*. The Genome Analysis Toolkit: a MapReduce framework for analyzing next-generation DNA sequencing data. *Genome Res*. Sep 2010;20(9):1297-303. doi:10.1101/gr.107524.110

6. Pais LS, Snow H, Weisburd B*, et al*. seqr: A web-based analysis and collaboration tool for rare disease genomics. *Hum Mutat*. Jun 2022;43(6):698-707. doi:10.1002/humu.24366

7. Monies D, Abouelhoda M, Assoum M*, et al*. Lessons Learned from Large-Scale, First-Tier Clinical Exome Sequencing in a Highly Consanguineous Population. *Am J Hum Genet*. Oct 3 2019;105(4):879. doi:10.1016/j.ajhg.2019.09.019

8. Matsuda T, Cepko CL. Controlled expression of transgenes introduced by in vivo electroporation. *Proc Natl Acad Sci U S A*. Jan 16 2007;104(3):1027-32. doi:10.1073/pnas.0610155104

9. Hand R, Polleux F. Neurogenin2 regulates the initial axon guidance of cortical pyramidal neurons projecting medially to the corpus callosum. *Neural Dev*. Aug 24 2011;6:30. doi:10.1186/1749-8104-6-30

10. Diebold ML, Fribourg S, Koch M, Metzger T, Romier C. Deciphering correct strategies for multiprotein complex assembly by co-expression: application to complexes as large as the histone octamer. *J Struct Biol*. Aug 2011;175(2):178-88. doi:10.1016/j.jsb.2011.02.001

11. Nguyen L, Besson A, Heng JI*, et al*. p27kip1 independently promotes neuronal differentiation and migration in the cerebral cortex. *Genes Dev*. Jun 1 2006;20(11):1511-24. doi:10.1101/gad.377106

12. Ramos-Morales E, Bayam E, Del-Pozo-Rodriguez J*, et al*. The structure of the mouse ADAT2/ADAT3 complex reveals the molecular basis for mammalian tRNA wobble adenosine-to-inosine deamination. *Nucleic Acids Res*. Jun 21 2021;49(11):6529-6548. doi:10.1093/nar/gkab436

13. Gerber AP, Keller W. An adenosine deaminase that generates inosine at the wobble position of tRNAs. *Science*. Nov 5 1999;286(5442):1146-9.

14. Godin JD, Thomas N, Laguesse S*, et al*. p27(Kip1) Is a Microtubule-Associated Protein that Promotes Microtubule Polymerization during Neuron Migration. *Dev Cell*. Oct 16 2012;23(4):729-44. doi:10.1016/j.devcel.2012.08.006

15. Laguesse S, Creppe C, Nedialkova DD*, et al*. A Dynamic Unfolded Protein Response Contributes to the Control of Cortical Neurogenesis. *Dev Cell*. Dec 7 2015;35(5):553-67. doi:10.1016/j.devcel.2015.11.005

16. Kabsch W. XDS. *Acta crystallographica Section D, Biological crystallography*. Feb 2010;66(Pt 2):125-32. doi:10.1107/s0907444909047337

17. Emsley P, Lohkamp B, Scott WG, Cowtan K. Features and development of Coot. *Acta crystallographica Section D, Biological crystallography*. Apr 2010;66(Pt 4):486-501. doi:10.1107/s0907444910007493

18. Liebschner D, Afonine PV, Baker ML*, et al*. Macromolecular structure determination using X-rays, neutrons and electrons: recent developments in Phenix. *Acta crystallographica Section D, Structural biology*. Oct 1 2019;75(Pt 10):861-877. doi:10.1107/s2059798319011471

19. Williams CJ, Headd JJ, Moriarty NW*, et al*. MolProbity: More and better reference data for improved all-atom structure validation. *Protein science : a publication of the Protein Society*. Jan 2018;27(1):293-315. doi:10.1002/pro.3330

20. Behrens A, Nedialkova DD. Experimental and computational workflow for the analysis of tRNA pools from eukaryotic cells by mim-tRNAseq. *STAR Protoc*. Sep 16 2022;3(3):101579. doi:10.1016/j.xpro.2022.101579

21. Behrens A, Rodschinka G, Nedialkova DD. High-resolution quantitative profiling of tRNA abundance and modification status in eukaryotes by mim-tRNAseq. *Mol Cell*. Apr 15 2021;81(8):1802-1815.e7. doi:10.1016/j.molcel.2021.01.028

22. Gao L, Behrens A, Rodschinka G*, et al*. Selective gene expression maintains human tRNA anticodon pools during differentiation. *Nature Cell Biology*. 2024/01/08 2024;doi:10.1038/s41556-023-01317-3

23. Chan PP, Lowe TM. GtRNAdb: a database of transfer RNA genes detected in genomic sequence. *Nucleic Acids Res*. Jan 2009;37(Database issue):D93-7. doi:10.1093/nar/gkn787

24. Jühling F, Mörl M, Hartmann RK, Sprinzl M, Stadler PF, Pütz J. tRNAdb 2009: compilation of tRNA sequences and tRNA genes. *Nucleic Acids Res*. Jan 2009;37(Database issue):D159-62. doi:10.1093/nar/gkn772

25. Kames J, Alexaki A, Holcomb DD*, et al*. TissueCoCoPUTs: Novel Human Tissue-Specific Codon and Codon-Pair Usage Tables Based on Differential Tissue Gene Expression. *J Mol Biol*. May 15 2020;432(11):3369-3378. doi:10.1016/j.jmb.2020.01.011

26. Drost HG, Paszkowski J. Biomartr: genomic data retrieval with R. *Bioinformatics*. Apr 15 2017;33(8):1216-1217. doi:10.1093/bioinformatics/btw821

27. Charif D, Thioulouse J, Lobry JR, Perriere G. Online synonymous codon usage analyses with the ade4 and seqinR packages. *Bioinformatics*. Feb 15 2005;21(4):545-7. doi:10.1093/bioinformatics/bti037
