# Supplementary material for: ADAT3 variants disrupt the activity of the ADAT tRNA deaminase complex and impair neuronal migration
Source: Brain. 2025 Mar 22;148(9):3407–21. doi: 10.1093/brain/awaf109 (PMC12404733; doi:10.1093/brain/awaf109)
Supplement: awaf109_Supplementary_Data [file awaf109_supplementary_data.zip › brain-2024-02790-File014.pdf]

J.D.P.R., R.L. and E.B. conceived and designed the experiments, performed the experiments, performed statistical analysis and analyzed the data related to cellular, and functional studies in mice. P.T. conceived and performed *in utero* electroporation, collected the mouse samples, processed the tissues and did immunostainings, took care of mouse colonies, coordinated *in vivo* experiments and provided technical assistance. E.Br. performed analysis of sequencing data and helped preparing the figures. H.R.V., M.V.G. and E.R-M. purified recombinant complexes and performed co-expression assays. N.S. helped with Western-blotting. H.R.V. and C.R. performed all the structural analysis. C.R. led the structural work and contributed to the writing of the manuscript. L.M. produced the tRNA, performed *in vitro* deamination assays, and analyzed the results. T.S-G. and L.D. designed and planned the in vitro deamination assays and analyzed the results. D.D.N. and T.B. performed tRNA sequencing and data analysis. G.V., E.M.E, A.K.L., M.O'L., M.C., N.M.O., M.B.T., A.E., Ma.A., M.M., L.S.P, R.C.Y., D.G., M.O.H., F.A., M.R.A., L.Z.S., O.A.A., R.A.K., G.O., A.R., Ha.A., He;A., E.T., Af.A., Mo.A., A.T., K.A., Am.A, Ai.A., M.K., B.S.A-A., W.E., N.A., A.O'D.L., J.E.N, J.G.G., C.A.W., N.E., F.S.A., L.A., N.E., L.S., S.S. followed-up the patients and families, provided the clinical and imaging data and/or contributed to the generation of whole-exome sequencing, bioinformatics tools and analysis of sequencing data. E.B. and J.D.G. conceived, coordinated and supervised the study and wrote the manuscript with contributions from all other authors.
